# Exploring non-target screening variability in unsupervised multivariate time trend analysis of LC-HRMS data

**DOI:** 10.1007/s00216-025-06225-z

**Published:** 2025-11-24

**Authors:** Reyhaneh Armin, Maryam Vosough, Torsten C. Schmidt

**Affiliations:** 1 Instrumental Analytical Chemistry, University of Duisburg-Essen, Universitätsstr. 5, 45141 Essen, Germany; 2 Water Management, Currenta GmbH & Co. OHG, 51368 Leverkusen, Germany; 3 Department of Clean Technologies, Chemistry and Chemical Engineering Research Center of Iran, P.O. Box 14335-186, Tehran, Iran; 4 Centre for Water and Environmental Research (ZWU), University of Duisburg-Essen, Universitätsstr. 2, 45141 Essen, Germany; 5 IWW Water Centre, Moritzstr. 26, 45476 Mülheim an Der Ruhr, Germany

**Keywords:** Non-target screening, Time trend analysis, SPCA, Unsupervised learning, LC-HRMS, Industrial wastewater

## Abstract

Non-target screening (NTS) using liquid chromatography-high-resolution mass spectrometry has become essential for uncovering unknown contaminants in complex matrices such as industrial wastewater. A key goal in these applications is detecting and interpreting time trends, especially spill-related events. The clarity of multivariate models depends on the quality of feature lists from various software tools. In this study, we evaluated five peak picking tools (MarkerView, MZmine3, XCMS, OpenMS, and SIRIUS) for unsupervised time trend exploration using sparse principal component analysis (SPCA). SPCA selects the most informative features per component, improving interpretability and reducing confounding variables. Two datasets were used: a controlled validation set of pooled wastewater samples with spiked target compounds exhibiting known profiles and a real-world dataset comprising 52 consecutive daily industrial wastewater samples. The first dataset facilitated analysis of tuning parameters with SPCA distinguishing spiking patterns associated with components, highlighting differences in feature/artifact prioritization across tools. Tools XCMS, MZmine3, and OpenMS showed higher consistency and were selected for next analysis. In the second phase, SPCA was combined with stratified bootstrapping (SBS-SPCA) to assess the reliability of trend detection, exemplified by specific targets. Five out of nine markers, showing more temporal persistency, were robustly detected across tools (selection frequency > 70%) under optimized tuning conditions. These findings indicate that interpretable, sparse models enhance marker detection in unsupervised settings and shed light on how software-driven feature structures impact multivariate outcomes in time-series NTS data. Such insights are especially pertinent for future high-throughput applications involving temporally dynamic exposure scenarios.

**Graphical abstract:**

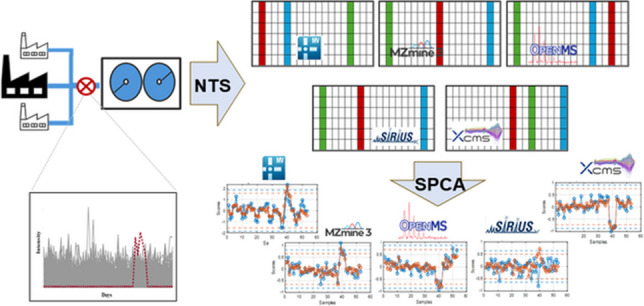

**Supplementary Information:**

The online version contains supplementary material available at 10.1007/s00216-025-06225-z.

## Introduction

Environmental emissions from various sources such as industrial wastewater have become a growing concern, particularly due to the release of numerous, often unidentified chemical substances into our aquatic systems. Comprehensive and continuous monitoring of industrial outlets via analytical techniques is critical for environmental safety and public health. Traditional methods such as gas chromatography-mass spectrometry (GC-MS) and tandem mass spectrometry (LC-MS/MS) have enabled effective analysis of known pollutants. However, these fall short in detecting unknown pollutants particularly in the case of chemical spills, leaving a gap in environmental monitoring.

Non-target screening (NTS) using liquid chromatography-high-resolution mass spectrometry (LC-HRMS) is proven to address this limitation by enabling the detection of a broad range of unknown or emerging compounds without requiring prior information [1–4]. Despite its demonstrated potential, the application of NTS on a regulatory basis remains a challenge. This is largely associated with the high complexity of the data generated, in addition to variability observed in data processing outcomes [5–7].

Feature extraction is a critical step in NTS and refers to the transformation of raw data into a manageable feature table. Based on the analytical question, the feature table is further processed with chemometrics or machine learning approaches. A range of software tools exists for the feature extraction process, all following a similar workflow but employing distinct mathematical and algorithmic approaches. Open-source options such as MZmine3, XCMS, OpenMS, and SIRIUS, along with vendor-specific tools like MarkerView, are widely used in the community [8–12].

Previous studies have demonstrated a lack of consensus across feature extraction software, resulting in discrepancies in the final results. For example, Hohrenk et al. [13] compared four commonly used software platforms and concluded that only 10% of top 100 features with the highest intensities overlapped. Guo and Huan [14] also identified major discrepancies between five commonly used software in metabolomic studies, and highlighted the critical role of chromatographic peak characteristics in influencing feature extraction outcomes. Schulze et al. [6] identified inconsistencies in the results of exploratory data analyses employing principal component analysis (PCA), which were related to the variability of feature tables generated by different peak picking algorithms within a spatiotemporal framework. In our recent study [15], we compared MZmine3 with the component-based regions of interest-multivariate curve resolution/alternating least squares (ROIMCR) [16] approach using mesocosm-derived LC-HRMS data from river water impacted by treated wastewater. Treatment and temporal effects were evaluated under controlled exposure using design-based and supervised multivariate models, revealing general agreement on main effects but clear differences in effect size estimates and feature prioritization. These discrepancies can lead to contrasting interpretations of environmental data, raising concerns about the consistency and reliability of NTS in regulatory assessments, and further highlights the importance of NTS workflow(s) selection and methodological comparisons across other contexts such as time-series studies, as demonstrated in the current work.

PCA is often applied to explore NTS data due to its utility in reducing dimensionality and highlighting variance patterns [7, 17–19]. This approach offers an effective method for data reduction, particularly useful for handling complex datasets through matrix factorization. PCA has several limitations that may make it difficult to retrieve the structure among variables. In particular, (i) it does not differentiate between unique and shared variances, and (ii) the components are linear combinations of all variables simultaneously [20–22]. Therefore, each component typically compresses the variance of several interdependent variables. The interpretation of high-dimensional data such as LC-HRMS data and loading contributions to latent components is greatly complicated by standard PCA approach [23, 24].

To overcome the limitations of standard PCA, such as the inclusion of all variables in each component and lack of variable selection, alternative approaches have been proposed to define a calibration algorithm for factorization that trades off variance explained and model simplicity. A subset of these techniques is known as sparse PCA methods (SPCA) [20–22] and group-wise PCA (GPCA) [25]. In SPCA, constraints are usually imposed by regularization terms that lead to a simple structure, so that the number of non-zero loads in a single PC is reduced by focusing on those that cause a variance, and imposing a simple structure to the loading vectors [26]. According to Zou et al. [22], SPCA can be defined by redesigning PCA as a regression-based optimization problem with ridge penalties. By incorporating L1 regularization (least absolute shrinkage and selection operator, LASSO) through the elastic net framework, this approach yields a modified PCA that produces sparse loadings, thereby focusing the model on a smaller subset of informative features. This not only enhances interpretability but also helps suppress noise from irrelevant or redundant variables. These properties are particularly beneficial in high-dimensional datasets, where clear linking between specific features and latent spaces is crucial for downstream analysis and marker prioritization. Given these properties, SPCA may be particularly relevant in NTS, where the aim is to explore complex, LC-HRMS datasets, such as those obtained from time-series studies, in a more interpretable and focused manner.

Time-series data play a crucial role in environmental monitoring by capturing temporal dynamics and trends. When combined with NTS, they offer valuable insights into the evolving chemical profiles of samples across both short- and long-term time scales. For instance, data from a riverbank filtration system have been used to investigate the occurrence of polar micropollutants over a 60-year travel period [27], while wastewater influent time-series data have been applied to prioritize and identify features of interest [23]. In the latter case, Spearman’s rank correlation served as a prefiltering step, followed by GPCA to cluster correlated features and reduce data complexity. However, the extent to which the choice of feature extraction tools influences the interpretation of time-series data, particularly in combination with unsupervised multivariate methods, remains an open question requiring further investigation.

The main objective of this study is the implementation of SPCA as an unsupervised learning method for the comprehensive evaluation of long-term temporal trends and feature prioritization in industrial wastewater, specifically targeting various spill types. We present an in-depth comparison of five feature extraction software workflows—MarkerView, MZmine3, and three PatRoon-integrated platforms (OpenMS, SIRIUS, and XCMS)—to determine how the choice of data processing pipeline influences SPCA outcomes and feature selection in the context of exploratory data analysis. The study employs two proof-of-concept datasets: first, replicated wastewater samples to compare model parameters, feature ranking and identify software pipelines with the highest consistency; second, developing a stratified bootstrapping-based SPCA (SBS-SPCA) to evaluate the interpretability and robustness of the SPCA models and feature selection in real wastewater time-series data. These findings may also inform future applications of SPCA in other high-throughput time-series datasets, where complexity and dimensionality pose similar analytical challenges.

## Materials and methods

### Chemicals

LC/MS grade methanol from Fisher Scientific (Geel, Belgium), Milli-Q ultra-pure water from Merck (Darmstadt, Germany), and LC/MS grade formic acid > 99% of HiPerSolv Chromanorm were used as an HPLC eluent system. Thirty-eight reference standards of high purity in addition to five deuterated internal standards (IS) were used in this work. A complete list of these can be found in the supporting information, in Tables S1 and S2, respectively.

### Studied samples

The samples were obtained from the wastewater treatment plant (WWTP) influent of an industrial park, comprising roughly 40 chemical plants. The influent samples are 24-h consecutive composite samples. Based on the metadata, the influent is dominated by (1) several HPLC-HRMS detectable chemicals with relatively high concentrations, and (2) a large number of chemicals present in lower quantities. The first category of substances necessitates the dilution of the influent samples. This procedure, however, further reduces the concentration of the second category of chemicals, posing a challenge to their detection. The presence of some chemicals is not constant, and their concentrations vary significantly with time, based on the production.

**Fig. 1:**
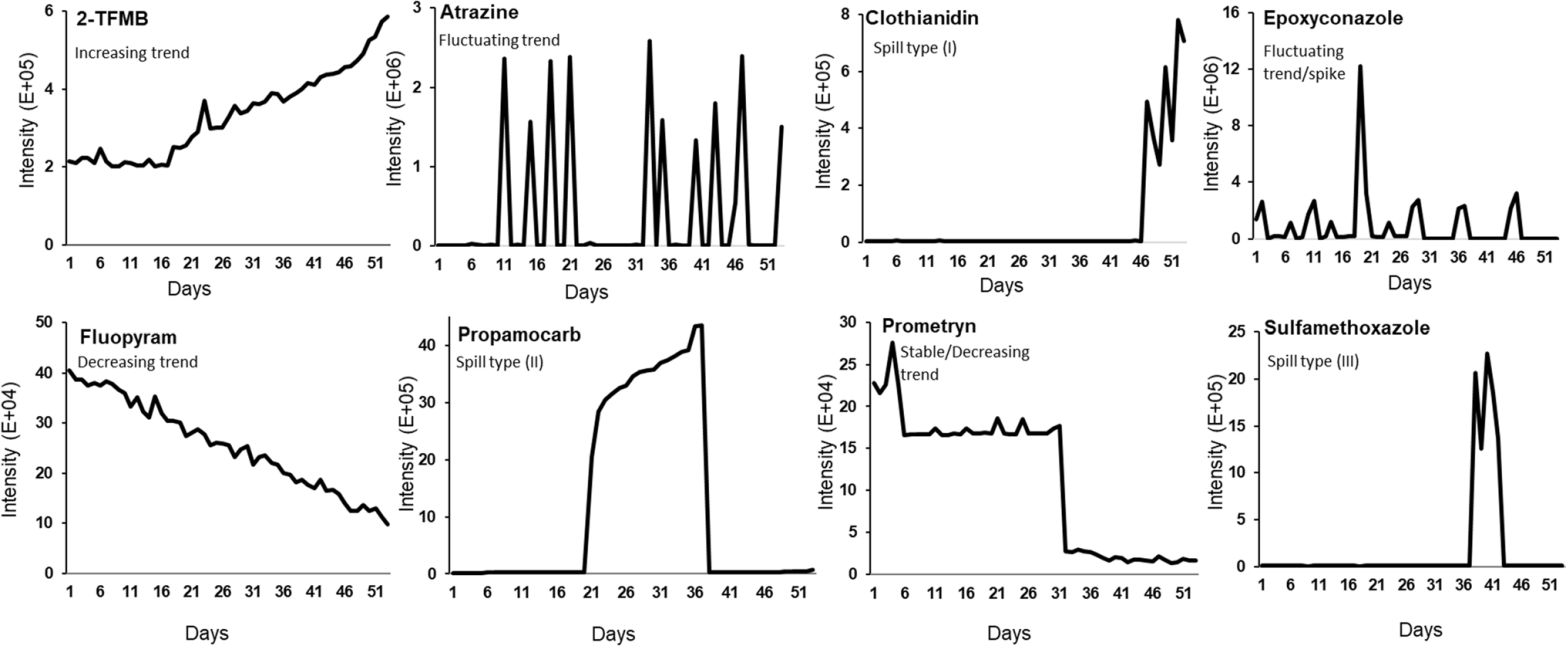
Reference intensity profiles for eight spiked targets in validation dataset I (53 replicate injections of a pooled wastewater matrix). The temporal patterns were derived from SCIEX OS and used to assess trend detection

In this study, different types of samples were considered, and QAQC samples were used for software parameter optimization and instrument performance monitoring. QC samples for software comparison was prepared by spiking 38 target compounds into eluent A to a final volume of 1 mL. Five levels of concentration (5–100 μg/L) were used. The 50 μg/L samples were initially used for optimizing software parameters, while the full concentration range supported evaluation of area-concentration linearity. All samples were spiked with four ISs prior to analysis and analyzed in triplicate. The limits of detection (LODs) and recovery rates are presented in Table S3. For method validation, two types of spiked wastewater samples were generated using standard target compounds that followed predefined temporal intensity patterns, serving as ground truth. The background matrix for validation set I, representing a matrix under controlled condition, were prepared by pooling of a batch of representative wastewater samples. Each of the 53 vials contained 100 μL of this matrix combined with 900 μL of eluent A. Each vial was then spiked with eight target compounds according to predefined intensity profiles, as detailed in Fig. 1.

**Fig. 2:**
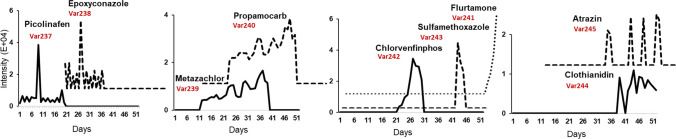
Reference intensity profiles for nine compounds spiked into validation dataset II (52 consecutive daily influent samples) to simulate real concentration fluctuations. Profiles were derived from SCIEX OS. The characterization of target profiles can be categorized as follows: left-sided spill profiles (Var237-238), wide spills (Var239-240), short-lived spills (Var241-243), right-sided spill (244), and episodic pattern (Var245)

The consistency of the matrix composition across these vials allowed focused evaluation of software sensitivity and trend detection under low-variability conditions. In contrast, validation dataset II aimed to reflect real-world complexity. It consisted of 52 individual 24-h composite influent wastewater samples collected on consecutive days. After centrifugation for 1 min at 2500 rpm (Centrifuge, 400 Function Line, Heraeus, Germany) and a tenfold dilution with Milli-Q water, each sample was spiked with five ISs and nine chemical standards at a concentration range of 8 μg/L to 350 μg/L, following temporal trends outlined in Fig. 2. The samples were once measured before the spiking to ensure the absence of these substances in the data matrix. All samples were measured in triplicates.

### LC-HRMS acquisition

A 5-μL aliquot of sample was injected into an Agilent 1290 Infinity HPLC system (Agilent Technologies, Waldbronn, Germany), into a C18 column and pre-column, namely the Trident cartridge (10 × 2.1 mm) and stainless steel filter (2 mm, 0.5 μm) connected to a Ultra Aqueous C18 (end-capped and modified with polar functional groups 100 mm × 2.1 mm, 3.0 μm particle size), both from Restek GmbH (Bad Homburg v. d. Höhe, Germany). The Agilent HPLC system was coupled to a x500R QTOF high-resolution mass spectrometer with a Turbo V ESI source (SCIEX GmbH, Darmstadt, Germany). The acquisition parameters for LC-HRMS are detailed in SI-1 (Table S4). The initial experiments involving parameter optimization were measured in positive and negative ionization modes. Analyses were then conducted in positive ionization mode due to the higher instrument's sensitivity to the spiked targets in validation samples and broader feature density in positive mode, focusing on software comparison rather than detailed influent composition and identification or elucidation of unknown compounds. The instrument was calibrated internally within the sequence every three measurements. Additionally, blank samples and QC samples were also measured multiple times throughout the sequence. The data acquisition was conducted with the software SCIEX OS (version 1.7.0, Framingham, USA).

### Preparing feature peak list

At first, a targeted approach via SCIEX OS software was performed on the QC samples to determine key metrics such as retention time (RT), intensity, and peak area of the reference standards. These values were utilized to optimize the parameters of the feature extraction software. To ensure accurate feature extraction, several critical parameters were systematically optimized within each software tool. These included configurations related to mass detection, extracted ion chromatogram (XIC) construction, componentization, alignment, and additional filtering steps. Each tool underwent multiple iterations of tuning to maximize recovery of target features in QC samples (see SI-2 and SI-4.1 for details). Two subsets of these reference chemicals were also spiked into wastewater samples to simulate temporal markers with varying patterns, enabling performance comparison of the feature extraction software through targeted diagnostics. Five different feature extraction software were examined in this study: (1) MarkerView (MV) (v. 1.3.1 Framingham, USA, SCIEX); (2) MZmine3 (MZM) (v. 3.2.8); (3) PatRoon (v.2.1.0), within which three algorithm packages, namely OpenMS (OMS), Sirius (SIR), and XCMS3 were utilized. PatRoon was operated in the Rstudio (2023.06.0, Boston, USA) environment. More information on each software is available in the SI-2. The detected feature intensities from each software were then imported into the MATLAB® (R2022a; MathWorks, Inc., Natick, MA) for further data processing. The final feature extraction parameters are described in detail in Tables S5 to S8.

### Data processing and unsupervised learning approach

Various data processing steps, including preprocessing and multivariate trend analysis, were conducted. For both validation sets, data normalization was performed using IS peak intensities. For the real sample dataset (dataset II), additional feature filtering and preprocessing was implemented. Highly spiky and uninformative features were removed using caret’s nearZeroVar (NZV) with freqCut = 95/5 and uniqueCut = 8, ensuring that spill patterns with at least 4 consecutive non-zero values across 52 time points were preserved, while filtering out features lacking meaningful temporal variability. In addition, log transformation and total abundance normalization (row-wise) were performed to stabilize variance and support consistency across QC samples in multivariate space [23, 28].

Procrustes analysis was used to assess similarity between datasets by aligning each software’s output to a reference matrix from SCIEX OS data using geometric transformations. Multivariate Exploratory Data Analysis (MEDA) plots were utilized to visualize relationships among variables within latent spaces (e.g., principal components) in high-dimensional data, and to help compare the correlation/association structure of the feature tables produced by different software tools. MEDA outperforms standard correlation maps by better suppressing noise in correlation estimates, reducing spurious associations [29]. PCA scores, loadings, and MEDA maps were computed using the MEDA toolbox (https://github.com/josecamachop/MEDA-Toolbox). SPCA analysis (SI-3) was performed via the SpaSM MATLAB toolbox (http://www2.imm.dtu.dk/projects/spasm/) [22, 30]. Prior to multivariate modeling, all data matrices were autoscaled. For the comparative evaluation of feature detection across software platforms on the validation dataset, 10 PCs were considered. A balance between L1 (LASSO) and L2 (Ridge) penalties was determined and empirically validated for each data matrix to maximize recovery of target loadings in validation data. Since each software output differed in the number of variables identified, L1 penalties were assessed based on the number of non-zero loadings (NZLs) in each PC, corresponding to a greed of L1 (0.6–0.9) and L2 regularization (0.1–100) values. The resulting settings allowed for consistent and interpretable comparison of extracted features. In the second part of the study, focused on NTS evaluation using real wastewater samples, we adapted a stratified bootstrapping procedure (500 replicates) with SPCA modeling to assess the robustness of feature selection under realistic conditions [31]. The stratified bootstrapped SPCA (SBS-SPCA) was performed across L1 and L2 parameters, using multiple PCs. In each bootstrap iteration, stratified resampling was applied to preserve the group structure of spiked target compounds (as five consecutive time points), and SPCA was fitted to the resampled data. Selection frequency was computed for each variable across bootstrap runs, and empirical *p*-values were estimated based on the distribution of stability values through a permutation test. Features consistently selected with high frequency of bootstrap percentage and *p*-values (< 0.05) were considered robust markers. Optimal parameter sets (LASSO sparsity, Ridge penalty) were then used for a final fixed-SPCA model to extract interpretable score plots, sparse loadings, and explained variance. The entire procedure was executed using a custom MATLAB script.

## Results and discussion

### Feature extraction and Procrustes analysis

Following parameter optimization for the validation dataset I, five feature peak matrices were generated, each representing 53 samples and varying in feature count: MarkerView (1315), XCMS (280), MZmine3 (315), OpenMS (248), and SIRIUS (166). MarkerView extracted the highest number of features, while SIRIUS extracted the fewest. Targeted analysis using SCIEX OS confirmed the consistent detection of the eight spiked compounds across all pool samples. All software tools, except SIRIUS, successfully captured these targets; SIRIUS failed to detect 2-TFMB, fluopyram, and prometryn. Manual review of feature lists revealed that most software detected key isotopes and adducts linked to the main ion ([M+H]^+^). ^37^Cl isotopes of atrazine, clothianidin, and epoxyconazole were present in the feature lists of all software with an exception of MZmine3. However, the ^37^Cl isotope of fluopyram was absent from the OpenMS and XCMS datasets. For all targets, the [M+Na]^+^ adduct was included in the feature lists of MarkerView resulting from an improper componentization. Additionally, several adducts and in-source fragments were also present in in this feature list, as reported before [23].

**Fig. 3:**
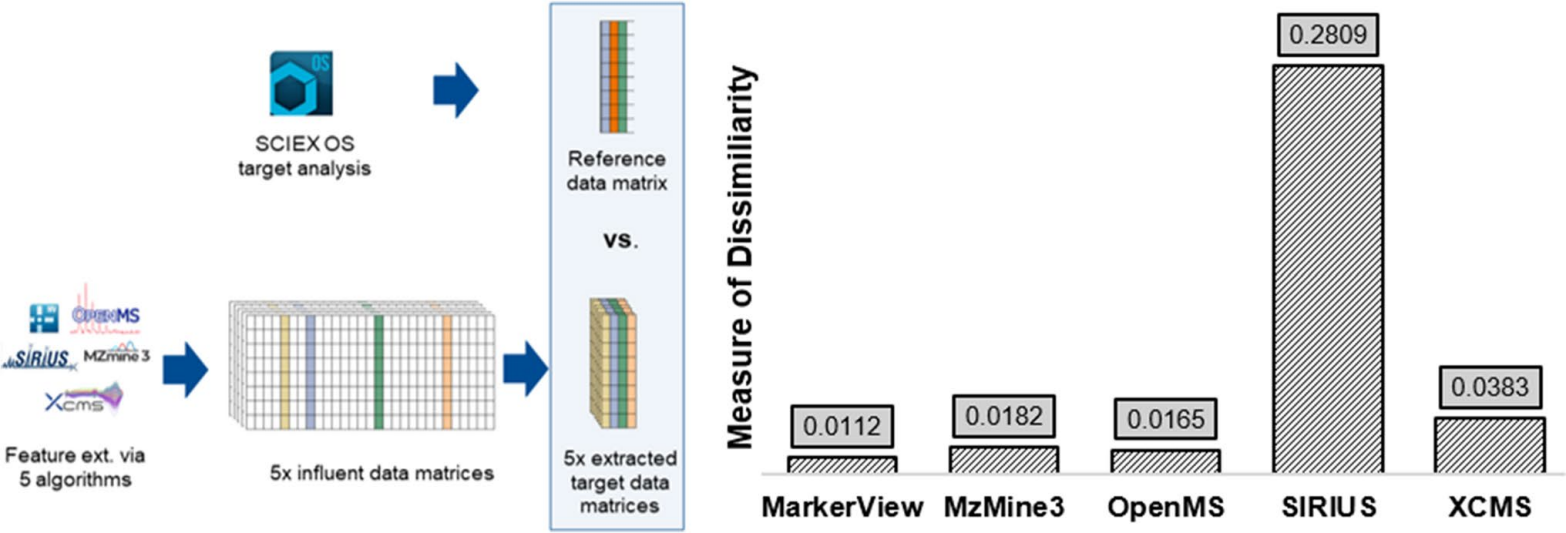
Procrustes analysis comparing each software-derived target matrix (MarkerView, MZmine3, OpenMS, SIRIUS, XCMS) to the SCIEX OS reference

Procrustes analysis was then used to evaluate the alignment between a reference data matrix, containing targeted [M+H]^+^ features from SCIEX OS (dstd), and feature profiles generated by five software tools across 53 pooled samples. As outlined in Fig. 3, the reference matrix was derived from targeted analysis in SCIEX OS, while the corresponding data matrices were constructed by manually extracting the same target compounds from each software’s feature list. Each matrix was individually compared to the reference. MarkerView exhibited the closest alignment (*d* = 0.0112), followed by OpenMS (0.0165) and MZmine3 (0.0182). XCMS showed greater dissimilarity (*d* = 0.0383), partly due to misalignments such as propamocarb duplication. SIRIUS demonstrated the highest dissimilarity (*d* = 0.2809), indicating the weakest concordance.

### Data exploration of validation dataset I

#### PCA

The initial data exploration was conducted using PCA for each feature list. The five scenarios demonstrate each algorithm’s influence on interpretations. The score and loading scatter plots for each software across PCs 1–4 (Figures S3–S6) show that the first two components mainly capture overall data variation, including instrument- or software-related signal fluctuations and potential sensitivity drifts not fully corrected by IS normalization. In practice, the incomplete IS-based correction was deliberately maintained to assess how different software tools respond to residual signal drift. In contrast, the third and fourth components primarily capture residual variation, including fluctuation and spill-like events introduced by the predefined spiking patterns (Figure S5), with interpretations varying depending on the MS software used (see SI-4.2 and Figure S7 for details).

#### SPCA

Principal components, however, are linear combinations of all variables in the matrix and are therefore difficult to label. In order to enhance the interpretability of scores/loading in a comparative analysis of provided feature lists and overcome limitations of PCA in inferences extracted on high-dimensional data, we suggest implementing sparse factorization methods, such as SPCA. In high-dimensional datasets, SPCA can reduce noise by isolating significant features and suppressing irrelevant ones. In the context of the current time-series LC-HRMS datasets, SPCA enhances component clarity in noisy datasets across various packages and assists in identifying key drivers of various time trends. Weak variable associations and unclear structure in MEDA maps reduce the effectiveness of sparse methods like GPCA or structured SPCA. This contradicts MarkerView, which exhibits clear feature clustering and makes it a suitable candidate for GPCA, as previously reported [23].

Another important superiority of SPCA is the possibility of detecting effective features with different spill structure in one subspace, which is not the case for GPCA, for example. For the current datasets, SPCA is a more suitable alternative as it applies sparsity constraints to select only the most relevant features, leading to a more concise model and improving interpretability without requiring predefined groups. Since the extracted features consist of unique precursors with/without adducts (or fragment), depending on the software, implementation of Elastic Net regularization which incorporates both LASSO (L1) and ridge (L2) penalties allows correlated variables to share influence, often entering the model together. This will allow control of the trade-off between variable selection and multicollinearity. Among SPCA formulations, we adopted the algorithm by Zou [22] and empirically balanced the L1 and L2 penalties for each data matrix to recover targeted features that capture discriminative patterns distinct from the general wastewater profile (see SI-3).

#### General trend observations

The final results of exploratory analysis of five data matrices using SPCA for first 10 PCs for target detection (true positives) under optimized penalty setting are provided in Table 1. Detection of target compounds start from PC2, since PC1 captures a general fluctuating pattern of wastewater replications.

**Table 1:** Results of SPCA for dataset I at two sparsity levels (L1= 70% and 90%) for the target substances, their adducts and isotopes (where available), extracted by the five feature extraction software. The first 10 PCs were considered and the PC at which the feature was assigned a non-zero loading has SPCA loading values within relevant subspaces have been presented in parenthesis. Bold loadings indicate top-ranked target features

	**2-TFMB**		**Atrazine**		**Clothianidin**		**Epoxyconazole**			**Fluopyram**		**Prometryn**		**Propamocarb**		**Sulfamethox**	
**Profile**	Increasing		Fluctuating		Spill (I)		Fluctuating/spiking			Decreasing		Stable/decreasing		Spill (II)		Spill (III)	
	**[M+H]**^**+**^	**[M+Na]**^**+**^	**[M+H]**^**+**^	**M**_**37**_**Cl**	**[M+H]**^**+**^	**M**_**37**_**Cl**	**[M+H]**^**+**^	**[M+Na]**^**+**^	**M**_**37**_**Cl**	**[M+H]**^**+**^	**[M+Na]**^**+**^	**[M+H]**^**+**^	**[M+Na]**^**+**^	**[M+H]**^**+**^	**[M+Na]**^**+**^	**[M+H]**^**+**^	**[M+Na]**^**+**^
**Feature extracted by:**	MV, OMS, MZM XCM	MV, OMS, MZM XCM	MV, OMS, MZM SIR XCM	MV, OMS MZM SIR XCM	MV, OMS, MZM XCM	MV OMS MZM XCM	MV, OMS, MZM SIR XCM	MV, OMS, MZM SIR XCM	MV, OMS, MZM SIR XCM	MV, OMS MZM XCM	MV, OMS MZM XCM	MV, OMS, MZM XCM	MV, OMS, MZM XCM	MV, OMS MZM SIR XCM	MV, OMS MZM SIR XCM	MV, OMS, MZM SIR XCM	MV, OMS, MZM SIR XCM
MV^b^ *Sp* = *70–90%, L2* = *1*	**p2 (0.16)**	**p2 (0.16)**	p10 (0.05)	p10 (0.01)	p6 (−0.02) **p7 (**−**0.10)** p9 (−0.05) p10 (−0.04)	p6 (−0.02) **p7 (**−**0.10)** p9 (−0.05) p10 (−0.04)	p6 (−0.03), p7 (0.003), p8 (0.001)	p6 (0.01)	**p6 (0.05)** P7 (0.02) P8 (0.02) (−0.01)	**p2 (**−**0.2)** p6 (0.02)	**p2 (**−**0.2)** p6 (0.02)	**p2 (**−**0.18)**	**p2 (**−**0.18)**	p3 (−0.11) p6 (−0.05) **p9 (0.14)** p10 (0.04)	p3 (−0.12) p6 (−0.05) **p9 (0.12)** p10 (0.04)	**p6 (**−**0.22)**	**p6 (**−**0.22)**
OMS *Sp* = *90%, L2* = *1*	**p2 (0.44)**	Grouped	**p5 (**−**0.55)**	**p5 (**−**0.55)**	**p4 (0.32)** p7 (−0.01)	**p4 (0.32)** p7 (−0.01)	**p6 (**−**0.30)**	Grouped	**p6 (**−**0.57)**	**p2 (**−**0.25)**	Grouped	**p2 (**−**0.26)**	Grouped	**p8 ** **(**−**0.42)** 9 (0.11)	Grouped	**p4 (**−**0.48)**	Grouped
MZM *Sp* = *90%, L2* = *1*	**p2 (**−**0.39)**	Grouped	p5 (−0.07) p7 (0.09)** p9 (**−**0.49)**	Grouped	**p5 (**−**0.29)**	Grouped	**p4 (0.28)**	Grouped	Grouped	**p2 (0.29)**	Grouped	**p2 (0.25)**	Grouped	p5 (0.26) **p6 (**−**0.33)**	Grouped	**p6 (0.40)**	Grouped
SIR *Sp* = *90% L2* = *10*	N.D.^d^	N.D.	**p5 (0.60)**	**p5 (0.60)**	N.D.	N.D.	**p4 (**−**0.51)** p9 (0.12)	Grouped	**p4 (**−**0.51)** p9 (0.12)	N.D.	N.D.	N.D.	N.D.	**p6 (**−**0.28)** p8 (0.03) p9 (−0.01)	Grouped	**p10 (0.66)**	Grouped
XCMS^c^ *Sp* = *70–90%, L2* = *1*	p2 (−0.18) **p2 (**−**0.25)**	Grouped	**p7 (**−**0.58)**	**p7 (**−**0.58)**	**p6 (**−**0.31)**	**p6 (**−**0.31)**	**p5 (**−**0.46)**	Grouped	**p5 (**−**0.46)**	p2 (0.21)	Grouped	p2 (0.18)	Grouped	p4 (0.11) **p6 (0.34)** p8 (0.09)	Grouped	**p4 (**−**0.52)**	Grouped

^a^Absolute SPCA loading values within relevant subspaces are presented in parenthesis. Bold loadings indicate top-ranked target features

^b^Up/downward trends obtained at L2 = 10 and sparsity (sp) = 90%. Clothianidin L2 = 10, sp = 70. Epoxyconazole and propamocarb L2 = 1 and sp = 70. Atrazin and sulfamethox L2 = 10 and sp = 90%

^c^Among up/downward trends, only 2-TFMB was periodized at sp = 70%, and retained as NZL at sp = 90%

^d^*N.D.* stands for non-detected target in the final feature list before SPCA

In this table, the non-detected features in the final feature list before implementing SPCA are marked. SPCA tuning parameters were optimized to ensure that each target in the validation dataset achieved NZLs with maximal absolute values within its respective subspace. Bolded loadings indicate target features ranked among the top 10 variables, selected as an ad hoc threshold for exploratory interpretation. When examining trend-specific recovery, we found that monotonic profiles were consistently captured by PC2 across all datasets (e.g., 2-TFMB), highlighting a degree of alignment in capturing the second dominant trend in the datasets. In contrast, compounds linked to fluctuating patterns or spill-type events showed less consistent alignment across software tools, although spill III was predominantly associated with PCs 4 or 6, indicating that such patterns can emerge more clearly within deeper latent spaces. Moreover, we see some trends in the data are detected in the subspace defined by a few PCs and not just one PC. This indicates that more complex chemical signatures (i.e., spill II) may span multiple PCs due to shared temporal patterns across several PCs. In addition, some PCs captured features related to more than one target. For instance, in OpenMS, PC4 contained markers for spills I and III, while in XCMS, PCs overlapped between spills I and II (PC6) and spills II and III (PC4). Generally, more than one spill signature is being captured in one specific PC, depending on the employed software.

**Fig. 4:**
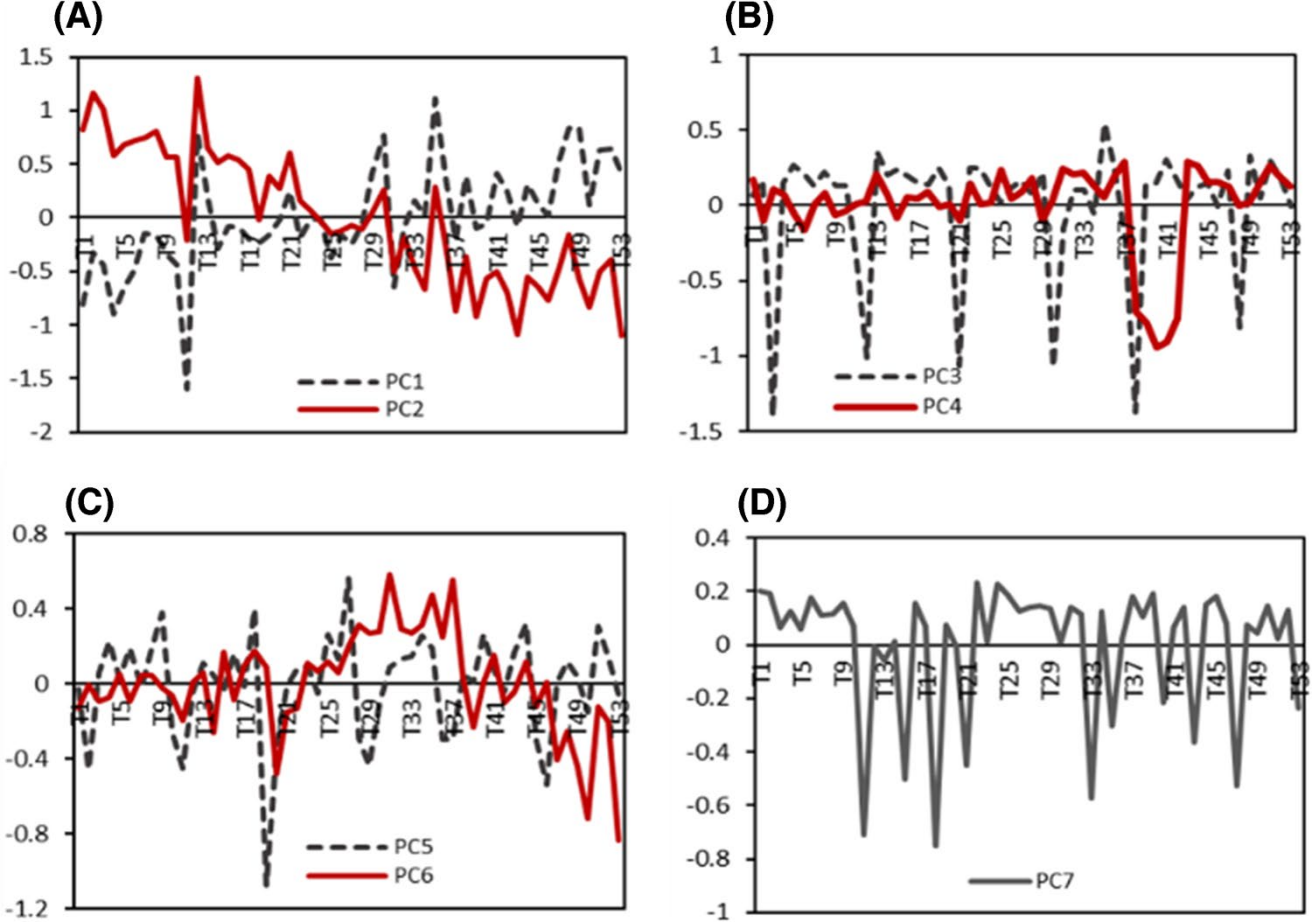
SPCA score plots from the first validation dataset (XCMS-derived) under L1= 90% sparsity and L2 = 1. **A** PC1 (4.3%) and PC2 (3.7%); **B** PC3 (3.3%) and PC4 (1.5%); **C** PC5 (1.4%) and PC6 (1.3%); and **D** PC7 (1.1%)

Figure 4 illustrates an example of SPCA score plots derived from the XCMS-processed data matrix, used to detect all spiked targets across seven PCs. Under a L1/L2 penalties 90%/1, these components collectively explain 16.6% of the total variance. Using XCMS, distinct temporal patterns were resolved, with upward/downward trends captured in PC2, spill type III in PC4, fluctuating/spike patterns in PC5, spills I and II in PC6, and general fluctuating/shifting trends in PC1 and PC7.

#### Exploring detected trends and similarities

Considering the three evaluation criteria—(i) top-ranked feature detection, (ii) PC number, and (iii) tuning parameters—no pair of tools met all simultaneously. Relaxing to (i)–(ii) yielded full agreement for monotonic (up/down) targets across tools except SIRIUS (targets absent). Under criterion (i) alone, propamocarb (spill II) and sulfamethoxazole (spill III) were consistently prioritized across all five tools. Overall, OpenMS, MZmine3, and XCMS showed greater internal consistency; with unified SPCA settings (L1 = 90% and L2 = 1), non-monotonic markers were reliably prioritized. When ignoring PC labels, the highest level of agreement is observed for OpenMS and MZmine3, both of which successfully recover all eight spiked target compounds across different latent spaces. Nonetheless, variance was partitioned differently across PC spaces (e.g., spill III in PC4 for OpenMS versus PC6 for MZmine3), indicating software-specific structuring of the same underlying signals (Tables S10, S11; Figure S8). XCMS required lower sparsity (L1 = 70%) to rank monotonic trends as leading, whereas MarkerView showed the largest variation in optimal tuning across trend types, and SIRIUS required stronger ridge (L2 = 10) to stabilize sparse solutions. Considering all evaluation criteria, Spill III showed the strongest cross-tool agreement, typically emerging in PC4 or PC6 under comparable penalties.

#### Feature ranking

**Fig. 5:**
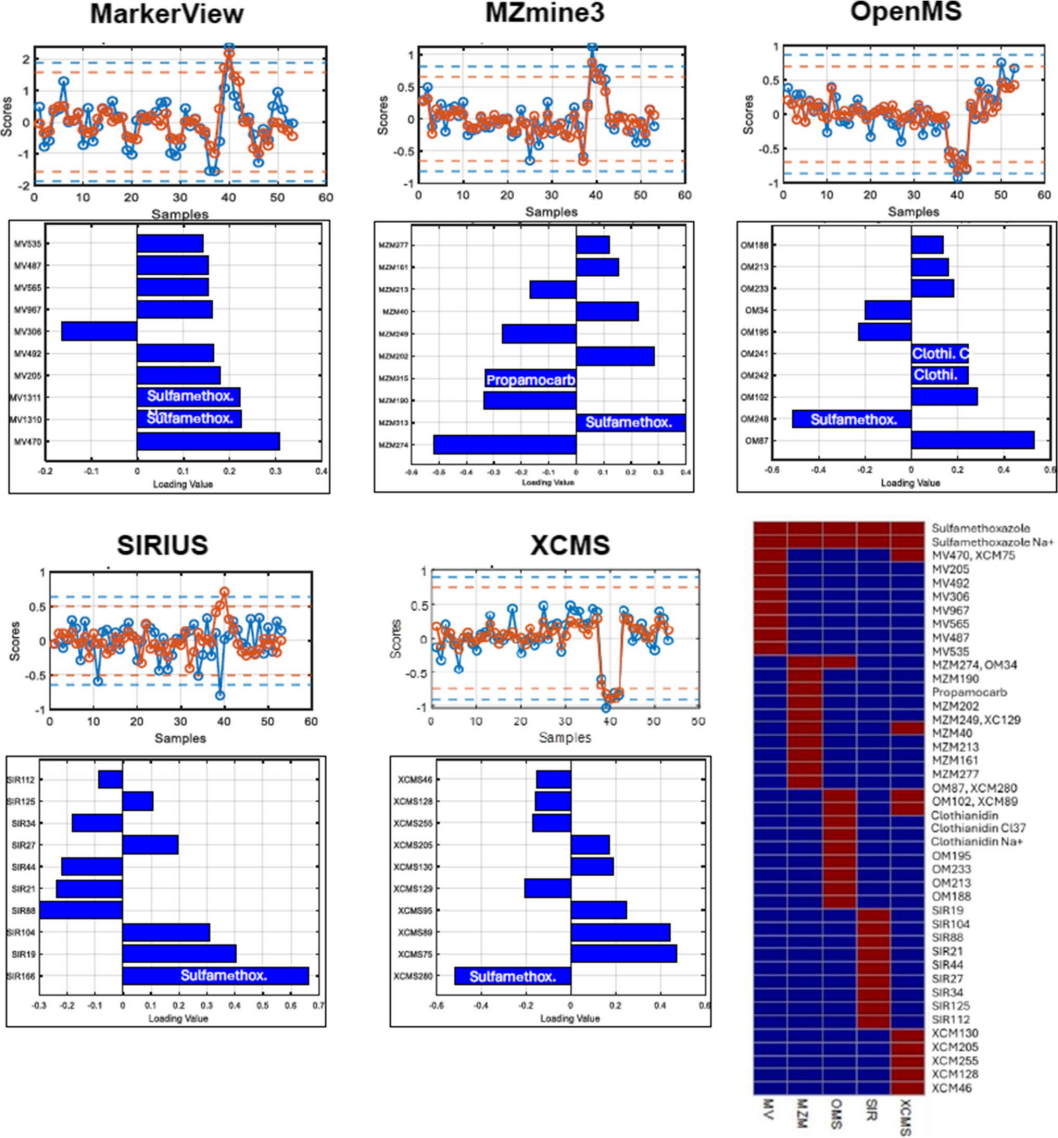
Comparative SPCA results for detecting spill III (sulfamethoxazole) across five LC-HRMS software tools (calculated with L2 = 10 for SIRIUS and L2 = 1 for the remaining). The score profiles under 90% (red) and 70% (blue) and top-ranked features under 90% sparsity (L1) are presented, highlighting sulfamethoxazole detection together with co-ranked targets and artifacts. Sulfamethoxazole-related features prioritized per tool are demonstrated in the binary heatmap

The features were ranked by absolute loadings within each component to identify pattern drivers, focusing on harmonized motifs (spill, monotonic) while noting potential matrix-derived false positives. Figure 5 presents SPCA scores alongside 99% control limits [32] highlighting temporal deviations related to spill type III. The corresponding loadings, capturing the transient trend with or without contributions from other spill types, are shown. The lower panels display the top-ranked variables under 90% sparsity, highlighting how each software tool prioritizes sulfamethoxazole-related signals within its data structure. The feature detection map reveals that only sulfamethoxazole was consistently identified across all platforms, while most other features—including matrix-related signals and potential false positives—were software-specific. Details for feature ranking for this spill pattern and monotonic trends are provided in SI (section 4.3, Figure S9). In the next step, to extract the elemental patterns of real industrial wastewater time-series samples, we focused on three software tools (OpenMS, MZmine3, and XCMS) based on their more consistent SPCA performance and targeted marker selections under unified tuning parameters, as demonstrated in the validation dataset.

### Data exploration of real wastewater samples: dataset II

This section investigates the multivariate structure of 52 real-world, composite (24-h) wastewater samples collected over consecutive days. Unlike the earlier phase, this dataset captures authentic temporal dynamics, marking a shift from the controlled conditions of the first experimental phase. PCA exploration of the selected data types reveals substantially greater dispersion and real temporal trends in influent wastewater samples, arguably indicating the presence of two main subclasses (see Figure S10). In the context of real industrial wastewater samples, sparsely detected true features and heightened matrix variation necessitate additional variable filtering and careful SPCA parameter tuning for robust feature stability assessment. A NZV filter was applied beforehand to remove features with mostly zeros and little variation. These features lack meaningful temporal patterns and distorted variance, potentially compromising model stability. NZV filtering reduced the number of features from 898 to 425 in MZmine3, from 535 to 425 in OpenMS, and from 2430 to 687 in XCMS. In the current experimental setup, a set of chemical markers representing sudden spills, evolving/disappearing patterns, and fluctuations (Fig. 2) was spiked into the samples to evaluate the performance of three data mining pipelines under varying tuning settings.

These events were modeled after real concentration variations observed in the metadata of the studied industrial wastewater influent. Such sudden increases often result from operational changes, cleaning procedures, or accidental releases from production facilities that feed the WWTP influent.

**Fig. 6:**
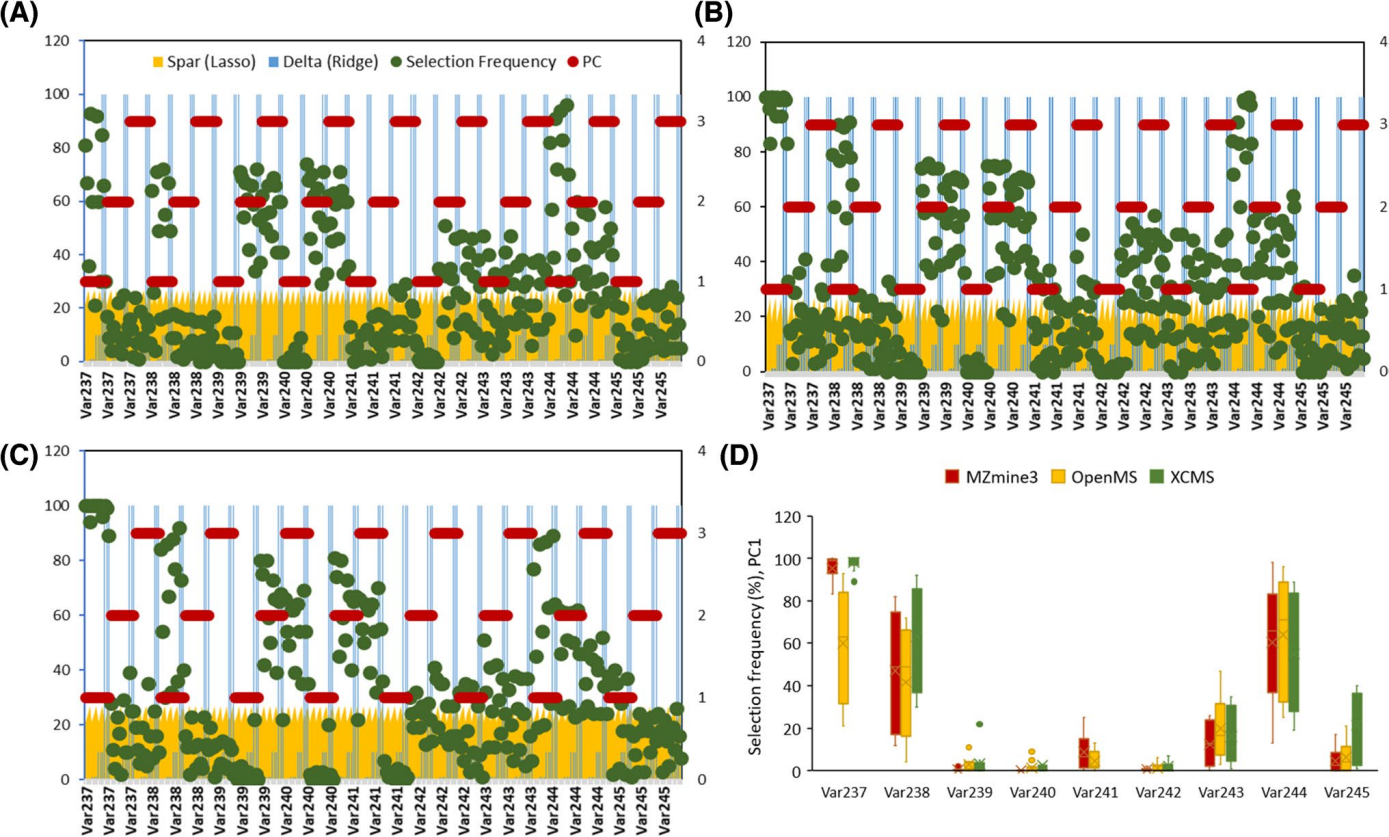
SBS-SPCA selection frequency profiles for target markers across PC1–PC3 and three software tools. **A** OpenMS, **B** MZmine3, **C** XCMS, and **D** is the span of frequency profiles for each marker on PC1. The marker indexes are unified according to OpenMS setting

To quantify the robustness of feature selection, we integrated a stratified bootstrapping with SPCA (SBS-SPCA) to the autoscaled datasets. Stratified bootstrapping ensured balanced resampling across temporal strata, preserving inherent structure while enabling stability analysis. For each software tool, SPCA was performed over 500 stratified bootstrap replicates, across a grid of sparsity (L1) and ridge (L2) penalties. For every replicate, variables with NZLs were recorded, allowing us to compute selection frequency for each feature. Figure S11 presents a comparative heatmap visualization summarizing the selection frequency of a defined panel of target markers for each software tool. A rapid assessment of the heatmaps revealed moderate to high stability for five target markers of picolinafen, epoxyconazole, metazachlor, propamocarb, and clothianidin (Var237–Var240, Var244), across primary latent variables in all three software tools. These markers consistently showed elevated selection frequencies under various regularization settings, indicating their robust contribution to the multivariate component structure. A higher resolution result under a restricted sparsity range (0.7–0.9) across PC1–PC3 is provided in Fig. 6.

**Fig. 7:**
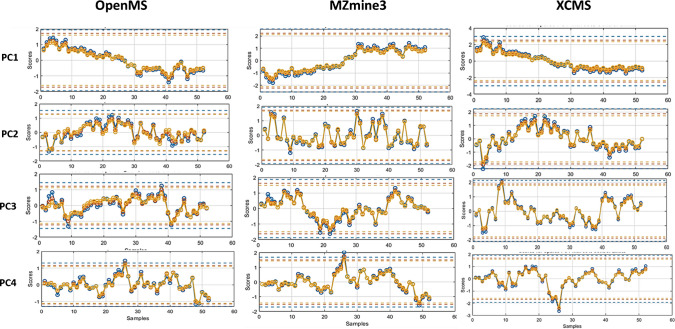
Sparse PC1–4 score plots generated using OpenMS, MZmine3, and XCMS software tools with harmonized parameters: L2 = 1, L1 = 0.6 (red line), and 0.7 (yellow line). Standard PCA scores are also displayed with blue lines

Panels A to C show variable-wise selection frequency results for OpenMS, MZmine3, and XCMS, respectively*.* As it is clear, three markers (Var237 and Var238 with left-sided spill profile and Var244 with right-sided spill profile) were consistently load on the first PC and markers Var239 and Var240 with patterns mimicking a “wide” spill load on PC2-3 subspace. In XCMS, picolinafen (Var237) was robustly captured in PC1 with 90–100% selection across the grid and in MZmine3 it showed ≥ 83% across settings. In OpenMS, this marker was stably detected only at sparsity < 0.8; increasing sparsity (e.g., to 0.9) favored competing matrix features and suppressed this target (Figure S12). A span of selection frequency for each marker on the first PC which is presented in Fig. 6D. To assess cross-software reliability, we quantified selection frequencies > 50% and > 70% (with unified variable indexing; *p* < 0.05) across 16 SBS-SPCA configurations, yielding 80,000 models over the first ten PCs. As shown in Figures S13 and S14, a general agreement emerges across software tools in identifying a core subset of target variables, which is more pronounced in early PC components. Additionally, SBS-SPCA configurations yielding selection frequencies above thresholds of 70% and 50% drop to zero beyond PCs 4 and 8. Nevertheless, specific divergences across tools can be observed depending on the evaluation criterion. When focusing solely on the capability of a selection frequency threshold > 70%, all three software tools consistently detect five markers characterized by distinct profiles—including left-sided spills (21–33% exposure), wide spills (52–54% exposure), and right-sided spills (25–27% exposure). However, if the proportion of marker selection across tuning parameters is also important, a pair-wise coherence is more obvious for most of the mentioned targets. Targets 237–240 showed pair-wise agreement across tools, while Var244 (clothianidin) achieved consistent cross-tool stability (mean proportion of selection frequency > 70%: 58 ± 7%) and was associated with PC1 in all datasets. By contrast, Var245 (atrazine; right-sided episodic, 13% exposure) exceeded the 70% threshold only in XCMS (6.2%) and only in PC1. In addition, all pipelines indicate that Var241–243 play a modest role in shaping the latent space, given their lower (< 70%) selection frequencies (see SI-4.3 for details). Following stability analysis, the most suitable and consensus-based parameter settings that satisfied stability criteria across all components were found to be L2 = 1 with sparsity values of 0.6 and 0.7. Using these optimized configurations, SPCA was re-applied to the full dataset. Figure 7 illustrates the PC1-4 score plots generated for different datasets using harmonized parameters. The plots illustrate both shared component (PC1) and tool-specific variations (PC2 for MZmine3). Higher components also reveal differences influenced by dataset-specific features. The variances in SPCA models show coherence and discrepancies compared to PCA, with improved harmonization at a lasso penalty of 70%, as shown in Figure S15.

To complement detection rates, we extended our prior approach by ranking target features based on absolute loadings within each sparse PC under unified SPCA settings. Table S12 compares target detection in all datasets using SPCA under two sparsity settings (L2 = 1; L1 = 0.7 and 0.6). Here, only the corresponding PCs showing the highest absolute loading values for each marker are shown. The following insights highlight both consistent patterns and notable divergences across the three software tools: (i) Features exhibiting broad spill trends (such as left/right-sided pulses or wide-spread exposures) demonstrated strong agreement across all platforms, yet ranked differently across tools. Variations in feature detection sensitivity, the size of the captured feature space, the prevalence of high-variance background signals, signal redundancy, episodic signal patterns (typical in industrial wastewater time-series data), and highly transient signals likely contribute to the observed divergences. These factors influence the shaping of temporal trends within PCs and the stability of variable selection. For example, inconsistent peak-picking of a true feature (across emission slot) can reduce its true detection frequency and loading strength. This is exemplified by picolinafen (Var237), which shows a reduced exposure rate (21%) in OpenMS compared to 32% in MZmine3 and 35% in XCMS, despite a true exposure 38%. This diminished its prominence despite relevance. (ii) Specific spill-like features characterized by short-lived or transient patterns with lower selection frequencies tend to exhibit greater variability across software tools. Their brief and abrupt appearances, mainly involving sharp intensity spikes confined to only a few time points and increased susceptibility to matrix effects, contribute to this sensitivity. Importantly, the challenges identified earlier for long-lasting features are even more prominent when dealing with transient features. For instance, sulfamethoxazole (Var243) is more confidently prioritized in PC7 (ranked 9th) with a selection frequency of 55% using OpenMS, likely due to its alignment with a time-specific spill pattern (see Figure S16). While such features can occasionally appear in lower PCs when their trends align with dominant components, their emergence as principal drivers is constrained by competition with more persistent, high-variance signals in earlier PCs. This highlights the importance of selection robustness, temporal alignment, and feature loading strength within PCs for reliably identifying informative markers, particularly during non-target feature prioritization where prior knowledge is lacking.

### Implementation of SBS-SPCA to non-targeted time-series data

SBS-SPCA framework is inherently unsupervised and readily transferable to real untargeted datasets without any prior target knowledge, supporting a data-driven-based prioritization scheme. Below, we outline how the same methodology can be extended and applied to fully untargeted time-series data: (1) The approach begins with applying stratified bootstrap-SPCA to the pre-filtered feature table, estimating NZL selection frequencies for all features across a grid of L1/L2 penalty combinations, (2) Features are then prioritized based on their robustness across bootstrap replicates and parameter settings. For example, those with selection frequency > 70% across multiple configurations and consistently within specific PCs are considered strong contributors, and prioritized for further exploration. (3) Identify L1/L2 combination(s) that consistently maximize feature stability across PCs, while retaining a substantial fraction of PCA variance. This enables definition of consensus tuning settings, adaptable to each dataset’s characteristics and the specific temporal question under investigation. (4) Time-resolved SPCA score trajectories (under optimized configurations) facilitate interpretation of dynamic patterns such as industrial discharges or spill-like events. Top-K features meeting high stability thresholds are further ranked based on their absolute loading values within each component. (5) To mitigate potential tool-specific selection biases (false negatives or underweighted features) and enhance coverage of robust signals, an additional feature aggregation step is introduced. This step retains features with high or moderate selection stability and strong loading within the same PC, as prioritized by at least one pair of software outputs. (6) The final feature set, derived through cross-tool prioritization and aggregation, can then be submitted for spectral database searches or in silico identification pipelines, forming a focused list of high-confidence, novel spill marker candidates. The workflow remains flexible: thresholds (e.g., in the 40–60% range for narrow/abrupt spills), number of retained PCs, L1/L2 penalty domains, and the choice between retaining all stable (NZL) features or a compact subset of high-loading markers can be tailored to matrix complexity and specific research goals. This provides a structured yet adaptable path for identifying markers aligned with specific spill types in complex environmental datasets. An illustrative example is provided in Figure S17, where the SBS-SPCA workflow was applied to non-targeted time-series dataset II processed by OpenMS and XCMS.

## Conclusion

In this study, the performance of five different feature extraction software, namely MarkerView, MZmine3, OpenMS, SIRIUS, and XCMS, were evaluated for non-target screening of data from untreated industrial wastewater. Using harmonized parameters across both controlled experiments and real-world time series, we evaluated how the structure of feature tables impacts subsequent multivariate analysis and interpretation. SPCA was introduced for the first time here as a central tool for unsupervised exploration of LC-HRMS data. SPCA improved interpretability by selecting a subset of informative variables within each latent component, allowing clearer identification of patterns such as spills, periodic fluctuations, and other shifts. In controlled experiments, spill-type events were captured more consistently by OpenMS, MZmine3, and XCMS, often in deeper latent components and under similar SPCA configurations. SPCA revealed that non-relevant matrix components and untreated spurious/artifact-derived signals can impair feature ranking and compete with genuine markers that drive specific abnormal trends, thereby distorting unsupervised trend analyses. This is particularly valuable in the real-world dataset, where high background complexity and matrix variability posed substantial challenges. By integrating SPCA with stratified bootstrapping, the study more precisely assessed the stability of feature selection, emphasizing the key markers that were consistently selected across different tools/PCs/sparsity conditions. Here, in spite of a shared and harmony for robust detection of markers (with persistent exposure patterns) across peak picking tools, the detection of short-lived trends shows more tool- and model tuning-dependent. Furthermore, even for features with high or moderate selection stability, discrepancies in loading-based rankings across tools persisted, reflecting differences in feature coverage, matrix effects, and how each tool represents the underlying signal space under sparsity constraints. While the controlled spiking experiments were primarily used to benchmark and tune the workflow, the developed SPCA-based approach remains fully applicable to genuine untargeted screenings, where it can highlight previously unknown yet temporally dynamic features. Our research indicates that utilizing at least two independent peak detection tools can significantly decrease false negatives especially associated with transient markers or sudden spills. Although this approach requires more computational resources, it enhances the reliability of marker identification in time-resolved studies. The proposed approach demonstrates broad applicability and generalizability to environmental NTS workflows and various types of longitudinal high-throughput datasets, especially in scenarios involving complex matrices and evolving temporal dynamics that require reliable and interpretable analysis.

## Supplementary Information

Below is the link to the electronic supplementary material.

Supplementary Material 1 List of chemical standards; LC-HRMS acquisition parameters; peak picking tools and parameter optimization; detailed processing settings for all algorithms; SPCA methodology; qualitative comparison of software outputs; multivariate analysis of validation and real wastewater datasets. (DOCX 6.66 MB)

## Data Availability

Data will be made available on request.
