# Supplementary material for: Exploring non-target screening variability in unsupervised multivariate time trend analysis of LC-HRMS data
Source: Anal Bioanal Chem. 2025 Nov 24;418(1):311–24. doi: 10.1007/s00216-025-06225-z (PMC12775120; doi:10.1007/s00216-025-06225-z)
Supplement: Supplementary file 1 — Supplementary Material 1 List of chemical standards; LC-HRMS acquisition parameters; peak picking tools and parameter optimization; detailed processing settings for all algorithms; SPCA methodology; qualitative comparison of software outputs; multivariate analysis of validation and real wastewater datasets. (DOCX 6.66 MB) [file 216_2025_6225_MOESM1_ESM.docx]

Electronic Supplementary Material

**Exploring Non-Target Screening Variability in Unsupervised Multivariate Time Trend Analysis of LC-HRMS Data**

**Reyhaneh Armin^1,2^, Maryam Vosough^1,3,4*^, Torsten C. Schmidt^1,4,5*^**

1. Instrumental Analytical Chemistry, University of Duisburg-Essen, Universitätsstr. 5, Essen 45141, Germany
2. Water Management, Currenta GmbH & Co. OHG, D-51368 Leverkusen, Germany
3. Department of Clean Technologies, Chemistry and Chemical Engineering Research Center of Iran, P.O. Box 14335-186,Tehran, Iran
4. Centre for Water and Environmental Research (ZWU), University of Duisburg-Essen, Universitätsstr. 2, Essen 45141, Germany
5. IWW Water Centre, Moritzstr. 26, Mülheim an der Ruhr 45476, Germany

**^*^**Corresponding Authors:

Maryam Vosough: maryam.vosough@uni-due.de

Torsten C. Schmidt: torsten.schmidt@uni-due.de

Table of Contents

[**1.** **Experimental** 3](#_Toc212455106)

[1.1 List of used standards 3](#_Toc212455107)

[1.2. LC-HRMS acquisition parameter 5](#_Toc212455108)

[**2.** **Peak picking software tools and the parameter optimization** 6](#_Toc212455109)

[2.1 MarkerView 6](#_Toc212455110)

[2.2 MZmine3 6](#_Toc212455111)

[2.3 PatRoon 7](#_Toc212455112)

[2.3.1 OpenMS 8](#_Toc212455113)

[2.3.2 SIRIUS 8](#_Toc212455114)

[2.3.3 XCMS 8](#_Toc212455115)

[2.4 Detailed processing parameters for all algorithms 9](#_Toc212455116)

[**3.** **Sparce principal component analysis (SPCA)** 13](#_Toc212455117)

[**4. Results** 14](#_Toc212455118)

[4.1 Qualitative assessment of algorithm performance 14](#_Toc212455119)

[4.2 Multivariate processing of validation dataset (I) 16](#_Toc212455120)

[4.3 Multivariate processing of real wastewater data: dataset (II) 22](#_Toc212455121)

[**5.** **References** 29](#_Toc212455122)

# **Experimental**

## 1.1 List of used standards

The following table summarizes the substances used in the QC samples for the optimization of the software. Some of these compounds were also spiked in the pool or influent samples. A list of the internal standards is also provided.

**Table S1**: List of components used in the quality control (QC) samples

| Compound | Formula | CAS | [M+H]^+^ | [M-H]^-^ | RT |
| --- | --- | --- | --- | --- | --- |
| 2-(Trifluoromethyl)benzamide | C8H6F3NO | 360-64-5 | 190.0474 |  | 6.81 |
| 3-Aminophenole | C6H7NO | 591-27-5 | 110.06 |  | 1.59 |
| 4-Chlorobenzoic Acid | C7H5ClO2 | 74-11-3 |  | 154.99 | 11.9 |
| 4-Methyl-benzotriazole | C7H7N3 | 29878-31-7 | 134.0964 |  | 13.35 |
| 4-Nitro-3-(trifluoromethyl)aniline | C7H5F3N2O2 | 393-11-3 | 207.0375 | 205.1595 | 17.71 |
| Alachlor | C14H20ClNO2 | 15972-60-8 | 270.1255 |  | 15.05 |
| AMTT | C5H5F3N4O | 124597-0 | 195.0485 |  | 7.92 |
| Atrazine | C8H14ClN5 | 1912-24-9 | 216.10105 |  | 13.25 |
| Azoxystrobin | C22H17N3O5 | 131860-33-8 | 404.1236 |  | 14.65 |
| Bentazon | C10H12N2O3S | 25057-89-0 | 241.0645 | 239.0496 | 11.07 |
| Benzamide Oxime | C7H8N2O | 613-92-3 | 137.0706 |  | 2.69 |
| Benzotriazole | C6H5N3 | 95-14-7 | 120.056 | 118.0412 | 6.7 |
| Bromyxynyl | C7H3Br2NO | 1689-84-5 |  | 273.8511 | 12.2 |
| Carbamazepine | C15H12N2O | 298-46-4 | 237.1018 |  | 12.66 |
| Chlorfenvinphos | C12H14Cl3O4P | 2005-03-27 | 358.9768 |  | 16.39 |
| Clothianidin | C6N5H8SO2Cl | 210880-92-5 | 250.016 | 248.0014 | 8.02 |
| Cyproconazlole | C15H18ClN3O | 94361-06-5 | 292.108 |  | 15.42 |
| Diclofenac Acid | C14H11Cl2NO2 | 15307-86-5 | 296.0239 | 294.0091 | 15.83 |
| Diphenyl sulfone | C12H10O2S | 127-63-9 | 219.0471 |  | 11.91 |
| Epoxyconazole | C17H13ClFN3O | 133855-98-8 | 330.08 |  | 16.19 |
| Fluopyram | C16H11ClF6N2O | 658066-35-4 | 397.0534 |  | 15.4 |
| Flurtamone | C18H14F3NO2 | 96525-23-4 | 334.1045 | 332.0903 | 14.46 |
| Imidacloprid | C9H10ClN5O2 | 138261-41-3 | 256.059 | 254.045 | 8.5 |
| MCPA | C9H9ClO3 | 94-74-6 |  | 199.017 | 12.93 |
| Metazachlor | C14H16ClN3O | 67129-08-2 | 278.1056 |  | 13.35 |
| N,N-Dimethyl-N'-phenylsulfamide | C8H12N2O2S | 4710-17-2 | 201.069 | 199.0546 | 9.34 |
| Naphthalene-2-sulfonic acid | C10H7SO3H | 120-18-3 |  | 207.0122 | 7.53 |
| Pentachlorophenol | C6HCl5O | 87-86-5 |  | 262.84 | 18.12 |
| Phathilic Anhydride | C8H4O3 | 85-44-9 | 149.0231 |  | 17.49 |
| Phenazone | C11H12N2O | 60-80-0 | 189.102 |  | 9.08 |
| Picolinafen | C19H12F4N2O2 | 137641-05-5 | 377.0907 | 375.0761 | 18.47 |
| Prometryn | C10H19N5S | 7287-19-6 | 242.1433 |  | 13.394 |
| Propamocarb | C9H20N2O2 | 24579-73-5 | 189.1597 |  | 4.15 |
| Sulcotrione | C14H13ClO5S | 99105-77-8 | 329.0232 | 327.0097 | 11.62 |
| Sulfamethaxazole | C10H11N3O3S | 723-46-6 | 254.059 | 252.0448 | 6.88 |
| Tebuconazole | C16H22ClN3O | 107534-96-3 | 308.1519 |  | 16.55 |
| Terbutryn | C10H19N5S | 886-50-0 |  |  |  |
| Tetraglyme | C10H22O5 | 143-24-8 | 223.0515 |  | 9.31 |

**Table S2:** List of internal standards

| Compound | Formula | CAS | [M+H]^+^ | [M-H]^-^ | RT |
| --- | --- | --- | --- | --- | --- |
| Bezafibrate D6 | C19D6H14ClNO4 | 1219802-74-0 | 368.1526 | 366.1384 | 14.24 |
| Diuron D6 | C9H4D6Cl2N2O | 1007536-67-5 | 239.062 | 237.0473 | 13.87 |
| Mecoprop D3 | C10D3H8ClO3 | 352431-15-3 |  | 216.051 | 14.48 |
| Metsulforon-Methyl D3 | C14H12D3N5O6S | 178928-70-6 | 385.1006 |  | 11.93 |
| imidacloprid D4 | C9D4H6ClN5O2 | 1015855-75-0 | 260.0846 | 258.0702 | 8.3 |

***Table S3:*** *Limits of detection (LOD) of the targets and their recovery rates in wastewater samples. A compound was considered successfully detected when a signal to noise ratio of 3:1 was achieved, and m/z (± 5 ppm) and R.T (± 0.1 min) were consistent across replicates*

| Compound | LOD (µg/L) | Recovery (%)* |
| --- | --- | --- |
| 2-TFMB | 1 | 84.4 |
| Atrazine | 0.1 | 68.9 |
| Chlorvenfinphos | 5.5 | 94.1 |
| Clothianidin | 2 | 74.4 |
| Epoxyconazole | 1 | 81.8 |
| Fluopyram | 1 | 76.8 |
| Flurtamone | 0.5 | 88.4 |
| Metazachlor | 1.5 | 41.6 |
| Picolinafen | 0.1 | 72.2 |
| Propamocarb | 6 | 67.5 |
| Prometryn | 0.5 | 65.8 |
| Sulfamethoxazole | 1 | 89.0 |

*Recoveries in concentrations of 50 µg/L on average.

## 1.2. LC-HRMS acquisition parameter

**Table S4**: Adjusted acquisition parameter the HPLC-HRMS system

| HPLC Parameter | HRMS Parameter |
| --- | --- |
| - HPLC system: Agilent 1290 Infinity - Eluent A: Milli-Q® H_2_O + 0.1% Formic Acid - Eluent B: MeOH + 0.1% Formic Acid - Gradient System: 0.5🡪1 min 🡪10% B, 20 min🡪 90% B, 26 min 🡪 90% B, 32 min 🡪 0% B - Flow: 0.300 mL/min - Injection volume: 5 μL - Column Temperature: 55 *°*C - Pre-Column: Restek^TM^ Trident cartridge (10 x 2.1 mm) equipped with 0.5 µm stainless-steel frit / filter - Column: Restek^TM^ Ultra Aqueous C18 column (100 mm x 2.1 mm, 3.0 μm). Reversed phase column with silica surface modified with polar groups. | - HRMS system: SCIEX x500R qTOF - Ion source: Turbo V Electrospray ion source - Source temperature: 450 *°*C - Source voltage: 5500 V for positive and  -4500 V for negative - Source gas 1: 50 psi - Source gas 2: 70 psi - Curtain gas:40 psi - Measured mass range: *m/z* 70-800 |

# **Peak picking software tools and the parameter optimization**

Before the application of the software for the influent data, the feature extraction settings were optimized as much as possible, to achieve the best results. Most of the software take a different approach towards extracting information from the raw data. With MZmine3 it is possible to double check the effect of the set parameters on the data processing. Additionally, one can go back to the previous step to fine-tune some settings if they are not optimal. Due to this reason, the first software which was tested and optimized was MZmine3. This software was the best option to “get to know” the data as it also offers visualization of the peaks/features at every step.

## MarkerView

MarkerView (MV) is a commercial software for the processing of LC-MS results generated by SCIEX instruments without the conversion of data to the .mzml format. After extracting the features, this interface enables the data analysis via statistical tools such as a PCA, PCVG and t-tests, making it a powerful tool in metabolomics and proteomics. It is user-friendly and does not require further platforms such as java, python or R. Within the scope of this work, MarkerView will be implemented only to extract features from the raw data to evaluate the peak picking performance.

Users of MarkerView are not given the option to manipulate many parameters to optimize the data processing workflow. While that accounts for the user friendliness of the software, it can also harm the quality of the generated data. The first step is peak picking and chromatogram development. The user defines the retention time window of interest and enters approximate LC peak width based on prior knowledge via markers or internal standards. At this stage, a predefined mass list can also be used to subtract background noise. In the second step, the user can remove C13 isotopes, align, normalize, and perform retention time correction of the data by using internal standards and by adjusting the mass and retention time tolerance. Finally, a feature list is created, which can either be exported or can be further processed via the in-built statistical tools of MarkerView. The chromatograms of each peak can also be viewed manually and individually, and the intensities of certain features can be plotted.

## 2.2 MZmine3

Mzmine3 (MZM) is one of the most widely used feature extraction software available and can be characterized as one of the “go to” open-source software for feature extraction [6]. The convenient applicability has rendered this interface highly popular across all non-target screening fields such as mainly -omics, environmental analysis, and food analysis. [7] first introduced MZmine2 in 2010, which was later updated the software to MZmine3 in 2023 [8]. It is important to note that for MZmine3 helpful and step by step tutorials are available.

For data processing in MZmine3, the data files from SCIEX instruments should previously be converted to .mzml format. Feature extraction via MZmine3 occurs in the following steps: 1) data import, 2) mass detection, 3) chromatogram building, 4) feature resolving, 5) isotope filtering, 6) alignment, 7) exporting the data. In some cases, there are more than one algorithm available for each data processing step. For example, in feature resolving several algorithms such as ADAP [6], CentWave [9] or Savitzky Golay resolves can be applied. Mzmine3 offers other optional data processing such as normalization, smoothing, gap filling if necessary. For each of the steps there are several parameters to be set. The interface allows the user to visualize and confirm the correctness of the parameters from one step to the next, and to redo a process for improvement.

The aligned feature lists play a crucial role in data filtering. They can be used to manually spot the adducts or further isotopes such as Cl or Br, while maintaining the metadata for each feature. Furthermore, by aligning the actual and the blank data, it is possible to remove the features resulting from background noise.

## 2.3 PatRoon

PatRoon, developed in 2021 by R. Helmus , is an open-source software based in *R* platform and offers a flexible workflow for non-target screening designed especially for environmental analysis. All data processing steps necessary for NTS are included within this user platform, from pretreatment with proteowizard extending to annotation of the found features and final data visualisation [10].

Within the environment of patroon, users can start processing their raw data with different algorithms including XCMS, OpenMS, Kpic2, SIRIUS and Envimass, perform componentization with packages such CAMERA and ramCluster, generate formulae and identify the features with further plug-ins such as GenForm and MetFrag and finally create reports. patRoon offers a “mix and match” when it comes to the algorithms. For instance, depending on the analytical question, it is possible to use XCMS as a feature finding tool and OpenMs to group the features.

Although patRoon operates in Rstudio and the users do need to have a very basic knowledge of this platform, it is not necessary to have a deep understanding of the R language. This gives the users the distinct advantage to use algorithms such as XCMS without encountering any complications and intricacies. Thanks to the GUI, all the data processing steps can be selected with a few clicks, and filtering options can be adjusted. It is of course possible to fine tune the parameters of each algorithm manually to get optimum results. For this purpose, patRoon offers the isotopologue parameter “IPO” package in Rstudio, with which it is possible to improve the parameters for data processing.

Since patRoon has eased the implementation of software for environmental samples, this platform is going to be used to evaluate the feature extraction ability of a few of these algorithms, namely OpenMS, SIRIUS and XCMS.

### 2.3.1 OpenMS

Not only in the patRoon environment, OpenMS (OMS) is a C++ based software very well designed for the visualization and analysis of LC-MS data available free of charge. This software has been implemented mainly for proteomics and metabolomics purposes [11]. The OpenMS tool and the TOPP tool (The OpenMS Proteomics Pipline) is built in patRoon for the feature finding and grouping stages, and each contain numerous parameters for optimization.

### 2.3.2 SIRIUS

SIRIUS (SIR) is a java based open-source software applied in the metabolomics field [12]. The advantage of SIRIUS against other software is the access to the “CSI:FingerID” the compound structure identification, which uses MS/MS data to elucidate the structure of metabolites.

Whilst the SIRIUS feature finding algorithm can be combined with other feature grouping algorithms such as xcms or openms, it is a perquisite for the grouping algorithm that the features are generated via the SIRIUS feature finding algorithm. In contrary to OpenMS and XCMS, patRoon users cannot influence the parameters set for these algorithms. In this work, the componentization of the SIRIUS generated feature lists were carried out with CAMERA, the parameter of which were the same as those used in XCMS.

### 2.3.3 XCMS

XCMS is one of the most frequently used algorithms for LC-MS data processing in many fields. For the XCMS version in R, there are unfortunately no graphical user interfaces available (except XCMS online), making XCMS difficult to work with. This means that users must have a good understanding of Rstudio in order to be able to use this software.

In patRoon, users can use XCMS to find features with various algorithms such as CentWave or Matchedfilter, and to group and align the features via PeakDensityParam and ObiwarpParam. Every algorithm employed is accompanied with many adjustable parameters. Whilst these contribute significantly to superior outcomes, it must be taken into consideration that their optimization can be time-consuming and highly complicated.

# Detailed processing parameters for all algorithms

The QC samples (50 μg/L) were used for parameter optimization with each of the software. The aim was to achieve the highest recall rates whilst keeping the false positives at minimum. After many iterations, the best parameters for our study was established for each software and are listed below in the following tables. No parameters were available for SIRIUS within the patRoon package.

**Table S5**: Feature extraction settings for MarkerView

| Feature Ext. Step | Parameter |
| --- | --- |
| Data Format | .wiff2 |
| Peak finding and  chromatogram building | Minimum RT: 1.20 min  Maximum RT: 23.00  Approximate LC width: 10 seconds  Minimum I in counts: 8  Chemical noise I multiplier: 2  Perform Background subtraction: Yes |
| Alignment and filtering | RT tolerance: 0.3 min  m/z tolerance: 7 ppm  max number of peaks: 100000  Remove peaks in < 3 samples: Yes  Isotope filtering: Yes |
| Normalization | Perform sample normalization based on ISTD |

**Table S6**: Feature extraction settings for MZmine3

| Feature Ext. Step | Parameter |
| --- | --- |
| Data Format | .mzml |
| Mass detection | *Filter*   - Scan number: - - Base Filtering Integer: - - Retention time: 1.2 – 23.0 min - MS level: 1 - Scan defintion: - - Polarity: +/- - Spectrum type: Any   *Mass detector*   - Centroid - Noise level = 1000 |
| ADAP Chromatogram builder | *Filter*   - Scan number: - - Base Filtering Integer: - - Retention time: 1.2 – 23.0 min - MS level: 1 - Scan defintion: - - Polarity: + - Spectrum type: Any   *Min group size in # of scans:* 8  *Group intensity threshold:* 1500  *Min highest intensity:* 2000  *m/z tolerance: 7 ppm* |
| Chromatogram Resolving (ADAP Resolver) | *S/N threshold: 10*  *S/N estimator: Intensity window SN*  *Min feature height: 1500*  *Coefficient/area threshold: 110*  *Peak duration range: 0.1 - 1*  *RT wavelet range: 0.01 – 0.1* |
| 13C Isotope filter | *m/z tolerance: 7 ppm*  *Retention time tolerance: 0.1 min*  *Maximum charge: 1*  *Representative isotope: Most intense* |
| Componentization (Feature Grouping) | *RT tolerance:* 0.1 min   - [M+Na-H] 21.9825 m/z - [M+K-H] 37.9559 m/z - [M+NH3] 17.0265 m/z - Cl-Isotope: 1.9971 m/z - Br-Isotope: 1.9980 m/z   *m/z tolerance:* 7 ppm  *Max relative adduct peak height:* 80% |
| Join aligner | *m/z tolerance: 7 ppm*  *Weight for m/z: 0.75*  *RT tolerance: 1 min*  *Weight for RT: 0.25* |
| Normalization | Standard Compound Normalizer |

**Table S7**: Feature extraction settings for OpenMS within patRoon

| Feature Ext. Step | Parameter |
| --- | --- |
| Data Format | .mzml |
| Mass detection (findFeaturesOpenMS) | - noiseThrInt = 1000, - chromSNR = 3, - chromFWHM = 5, - mzPPM = 7, - reEstimateMTSD = TRUE, - traceTermCriterion = "sample_rate", - traceTermOutliers = 5, - minSampleRate = 0.5, - minTraceLength = 3, - maxTraceLength = -1, - widthFiltering = "fixed", - minFWHM = 5, - maxFWHM = 30, - traceSNRFiltering = FALSE, - localRTRange = 10, - localMZRange = 6.5, - isotopeFilteringModel = "metabolites (5% RMS)", - MZScoring13C = FALSE, - useSmoothedInts = TRUE, - extraOpts = NULL, - intSearchRTWindow = 3, - useFFMIntensities = FALSE, - verbose = TRUE |
| Grouping and alignment  (groupFeaturesOpenMS) | - rtalign = TRUE, - QT = FALSE, - maxAlignRT = 15, - maxAlignMZ = 0.002, - maxGroupRT = 15, - maxGroupMZ = 0.002, - extraOptsRT = NULL, - extraOptsGroup = NULL, - verbose = TRUE |
| Componentization  (generateComponents OpenMS) | - ionization = NEGATIVE/POSTIVE, - chargeMin = 1, - chargeMax = 1, - chargeSpan = 3, - qTry = "heuristic", - potentialAdducts = NULL, - minRTOverlap = 0.5, - retWindow = 1, - absMzDev = 0.002, - minSize = 2, - relMinAdductAbundance = 0.75, - adductConflictsUsePref = TRUE, |
| Filtering (Filter) | - blankthreshold = 5 - removalBlanks = True - RTrange = 80-1380 S - mzRange=80-800 |
| Normalization (normINTS) | - featNorm = "istd" - adduct= "[M+H]+" - ISTDRTwindow = 30 - ISTDMZWindow=50 - minISTDs = 2 |

**Table S8**: Feature extraction settings for XCMS within patRoon

| Feature Ext. Step | Parameter |
| --- | --- |
| Data Format | .mzml |
| Mass detection (findFeaturesXCMS3)  (CentWave) | - ppm = 7, - peakwidth = c(5, 30), - snthresh = 10, - prefilter = c(3, 100), - mzCenterFun = "wMean", - integrate = 1L, - mzdiff = -0.001, - fitgauss = FALSE, - noise = 800, - verboseColumns = FALSE, - roiList = list(), - firstBaselineCheck = TRUE, - roiScales = numeric(), - extendLengthMSW = FALSE |
| Grouping and alignment  (groupFeaturesXCMS3)  (PeakDensity, Obiwarp) | Grouping:   - bw = 30, - minFraction = 0.5, - minSamples = 1, - binSize = 0.25, - maxFeatures = 50   Alignment   - binSize = 1, - centerSample = integer - response = 1L, - distFun = "cor_opt", - gapInit = 0.2 - gapExtend = 0.8 - factorDiag = 2, - factorGap = 1, - localAlignment = FALSE, - initPenalty = 0, - subset = integer, - subsetAdjust = c("average", "previous") |
| Componentization  (generateComponents CAMERA) | - ionization = Positive/Negative - onlyIsotopes = FALSE, - minSize = 4, - relMinReplicates = 0.5, - extraOpts = NULL |
| Filtering (Filter) | - blankthreshold = 5 - removalBlanks = True - RTrange = 80-1380 S - mzRange=80-800 |
| Normalization (normINTS) | - featNorm = "istd" - adduct= "[M+H]+" - ISTDRTwindow = 30 - ISTDMZWindow=50 - minISTDs = 2 |

# **Sparce principal component analysis (SPCA)**

Over the years, principal component analysis (PCA) has been widely used to interpret NTS data [1]. In this model, PCs are linear combinations of all variables, making the interpretation of the results challenging, especially with high dimensional data. Sparse PCA (SPCA) enhances data interpretability by identifying a smaller subset of significant variable which accounts for the highest variation in the data. By incorporating sparsity through methods such as LASSO (Least Absolute Shrinkage and Selection Operator) as introduced by Zou et al. [2], SPCA nullifies many of the coefficients to zero. This ensures that each PC is influenced by only a limited subset of variables. Zou et al. introduced SPCA based on the following:

$$\left( \hat{W},\hat{P} \right)=\arg\min_{W,P} \|X-XWP^{\top}{\|}_{F}^{2}+\sum_{k=1}^{K} \lambda\parallel w_{k}\parallel_{2}+\sum_{k=1}^{K} \lambda_{1,k}\parallel w_{k}\parallel_{1}$$

In this formulation, $X$ is the original data matrix, $W$ represents the weights or loadings, and $P$ is an orthogonal matrix that defines the directions of the principal components. The term $\|X-XWP^{\top}{\|}_{F}^{2}$ measures the difference between the original data and its approximation using the sparse components. The inclusion of the penalties $\lambda\parallel w_{k}\parallel_{2}$ (Ridge) and $\lambda_{1,k}\parallel w_{k}\parallel_{1}$ (Lasso) encourages a balance between capturing variance and inducing sparsity in the weights $W ADDIN CITAVI.PLACEHOLDER$[3, 2]. The process involves an alternating minimization approach, where $W$ and $P$ are updated iteratively. With $P$ fixed, $W$ is optimized using elastic net regression, which combines Ridge and Lasso penalties to ensure both stability and sparsity in the solution. Conversely, with $W$ fixed, $P$ is updated to align with the current component scores, solving an orthogonal Procrustes problem. This method enables SPCA to effectively isolate and identify a small subset of variables that contribute most to the variation in a complex dataset.

The primary advantage of SPCA lies in its ability to enhance interpretability of the principal components by zeroing out less important features, making it particularly valuable for analyzing complex data such as environmental samples. In such cases, a few key pollutants or variables may drive most of the variance, and SPCA can pinpoint these crucial factors amidst thousands of measurements. This capability makes SPCA a powerful tool for identifying underlying patterns and significant drivers of variability, providing insights that are often missed in traditional PCA. This model has found diverse applications in bioinformatics, finance, and environmental monitoring [3–5].

# **4.** **Results**

### 4.1 Qualitative assessment of algorithm performance

**Parameter Optimization**

The recall performance of each software tool was assessed across five concentration levels, with detailed results for the lowest (C1) and highest (C5) levels presented in Table S9. The recall rates in this case is calculated based on the extraction of “true features”. Here, we define true features as the one representative feature for a target substance, which is in our case the proton adduct of a target. (Whether the [M+H]+ or [M-H]-were the representative features was examined prior via targeted analysis, with SCIEX OS.) False positives on the other hand are defined as the other types of signals characterized as a feature, for example noise/artifact signals or even other adducts such as [M+Na]+ which are present in, due to an incomplete componentization.

Despite parameter optimization, all tools faced challenges at the lowest concentration (C1), mainly due to poor peak shapes and low signal intensities. MarkerView extracted the most true features at C1 in both ionization modes but also reported the highest rate of false positives. SIRIUS showed the weakest recall, retrieving only half of the targets. At C5, all software except SIRIUS successfully extracted all targets in negative mode. Notably, none of the tools (except MarkerView) detected 3-Aminophenol largely due to low-intensity signals being filtered out during chromatogram construction (Figure S1).

**Table S9**: Performance of the software following parameter optimization for the lowest and highest concentration level of QC samples. ISTDs were also taken into consideration

|  | **Lowest Concentration Level C1** | | | | | | **Highest Concentration Level C5** | | | | |
| --- | --- | --- | --- | --- | --- | --- | --- | --- | --- | --- | --- |
|  | MV | MZM3 | OMS | Sirius | XCMS | MV | | MZM3 | OMS | Sirius | XCMS |
| **Recall rate (%) of true features extracted in positive mode  (overall 38)** | 84.2 | 68.4 | 68.4 | 57.8 | 78.9 | 94.7 | | 89.4 | 94.7 | 84.2 | 89.4 |
| **Recall rate (%) of true features extracted in negative mode  (overall 19)** | 61.8 | 36.7 | 33.3 | 44.0 | 73.5 | 74.2 | | 54.0 | 48.4 | 40.8 | 66.3 |


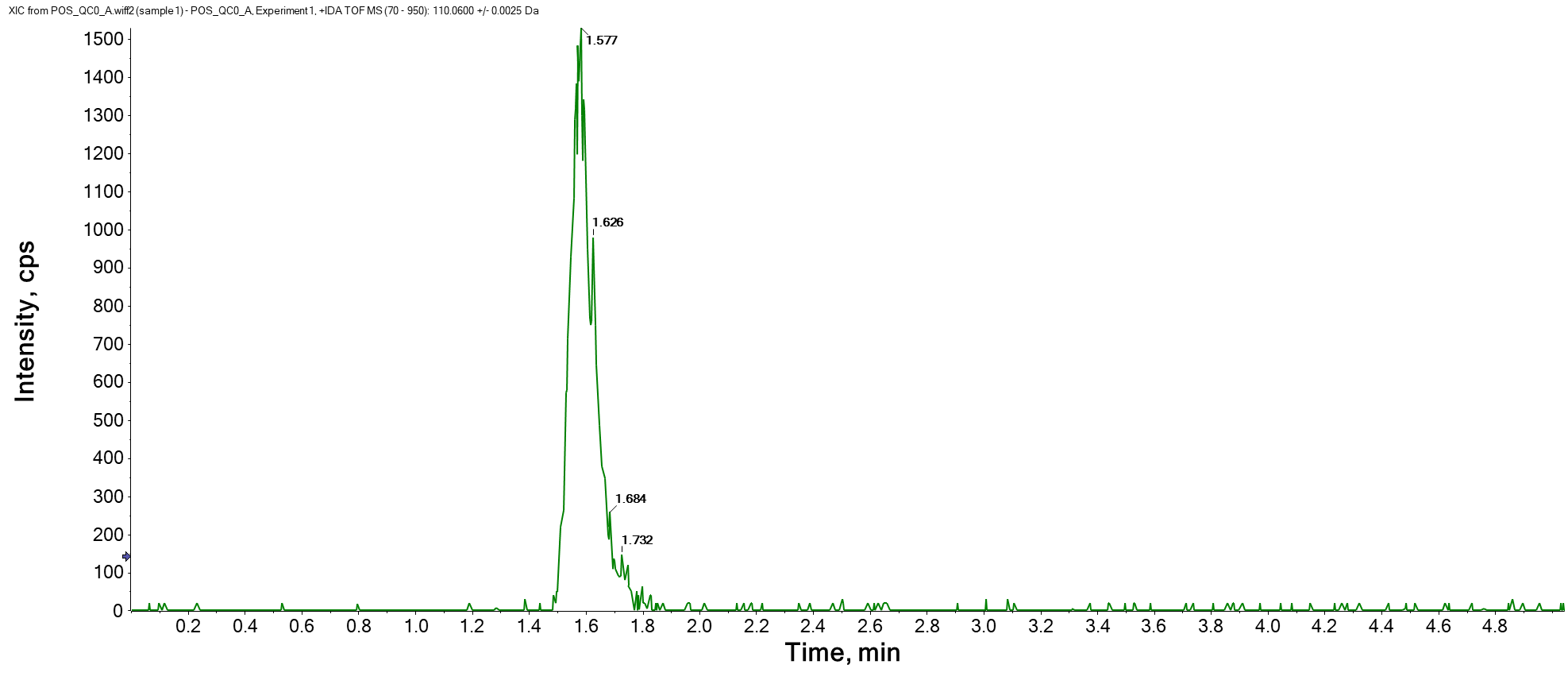


**Figure S1**: XIC of 3-Aminophenol, not properly constructed by any algorithm other than MarkerView. This resulted in the absence of 3-Aminophenol from all feature lists.

**Linearity**


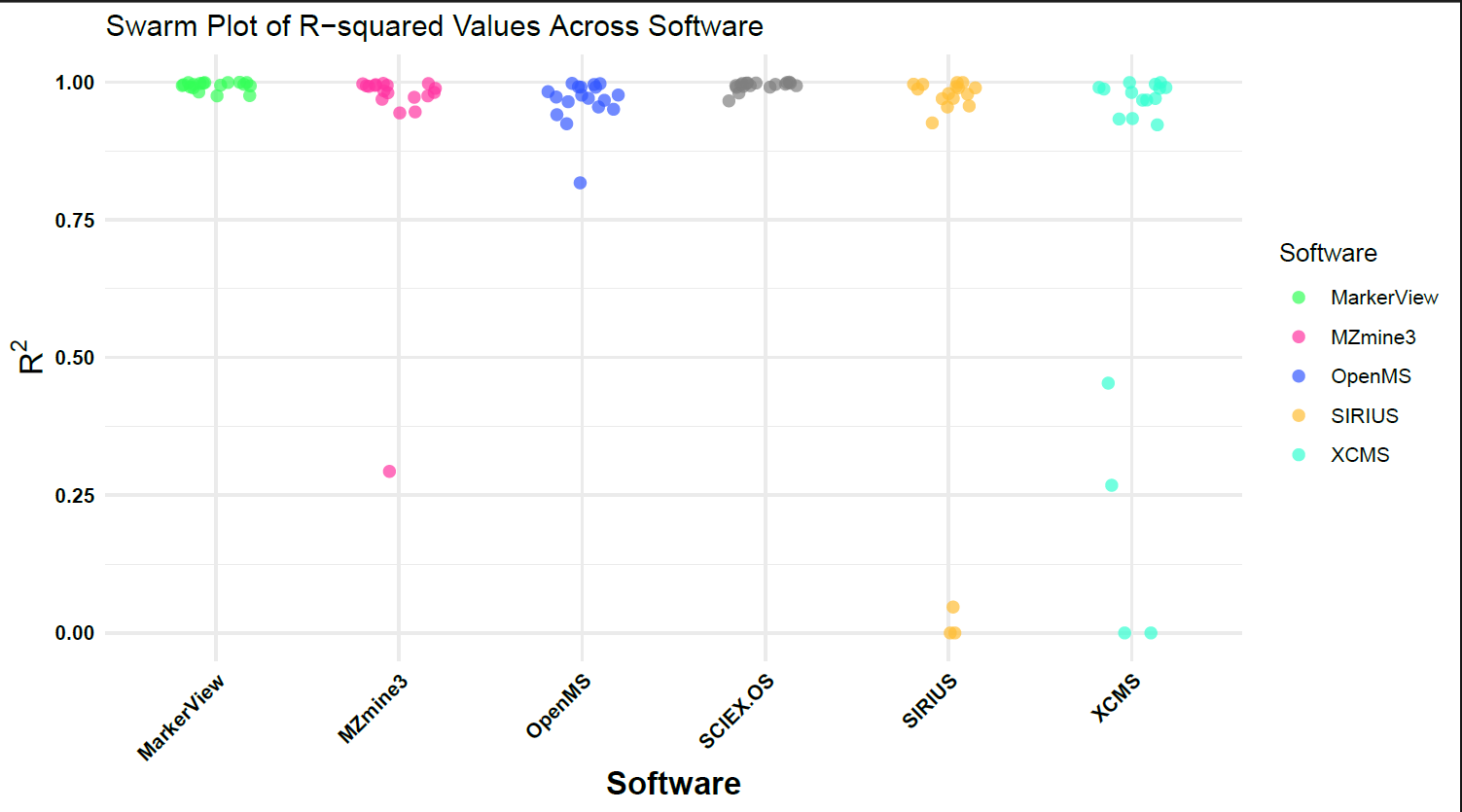
Additionally, linearity of the five sofware was assessed using 18 target compounds that showed a high correlation coefficient (R² ≥ 0.95) with the reference software, SCIEX OS. While most tools showed good linearity, SIRIUS and XCMS had inconsistent performance, often missing calibration points. In contrast, MarkerView, MZmine3, and OpenMS exhibited strong, consistent linear responses, with R² values closely clustered near 1.0, indicating accurate intensity quantification (Figure S2).

**Figure S2**: Distribution of the correlation coefficients (R^2^) of the constructed calibration curve for 18 targets, based on the intensity values of the 5 examined software and the reference software SCIEX OS

## 4.2 Multivariate processing of validation dataset (I)

Following PCA on each feature list (Figures S3-S6), the cumulative variance explained by the first ten principal components differed markedly—85% for MarkerView, versus 53% for XCMS, 47% for OpenMS, 56% for MZmine3, and 57% for SIRIUS.The notably higher variance captured by MarkerView show substantial divergence in its data-processing approach relative to the other platforms. To prevent the mapping of spurious correlations between features, the association structure of datasets was analyzed using MEDA plots. MEDA maps using the first 10 PCs reveals notable differences between the software in terms of correlation structure (Figure S7). MarkerView, lacking a componentization step, exhibits a dense correlation structure indicative of increased redundancy, including isotopologues, adducts, and fragment ions. Since a large proportion of wastewater-related features share the correlation pattern, they dominate the first PC with 42% of explained variance, shaping the overall trend of the validation set. Redundant peaks increase complexity, computational cost, and are affected by background noise, complicating data analysis. In MarkerView data, however, some unusual trends may appear in the lower PC subspace because of numerous interrelated features. Exploration of PC3-4 score plot (Figure S5) shows the sampling points 22-27 contributing to spill pattern (II) (Figure 1), which is only detected by MarkerView. Figure S6 displays loading plots indicating that the spill trend is closely linked to certain variables like 985, 1044, 1071, 1065, and 1313-1314.

**Figure S3**: Scatter plots of PC scores (PCs 1-2) for data matrices retrieved from the five feature extraction tools, for the validation dataset.

**MV**

**MZM**

**OMS**

**SIR**

**XCMS**

**Figure S4**: Scatter plots of PC loadings (PCs 1-2) for data matrices retrieved from the five feature extraction tools, for the validation dataset.

**SIR**

**OMS**

**MZM**

**MV**

**XCMS**

**MZM**

**OMS**

**MV**

**Figure S5:** Scatter plots of PC scores (PCs 3-4) for data matrices retrieved from the five feature extraction tools, for the validation dataset. For MarkerView, the calculated PC scores (PC3) for the 53 samples are also presented.

**MV**

**XCMS**

**SIR**

**MZM**

**OMS**

**MV**

**SIR**

**XCMS**

**Figure S6**: Scatter plots of PC loadings (PCs 3-4) for data matrices retrieved from the five feature extraction tools, for the validation dataset.


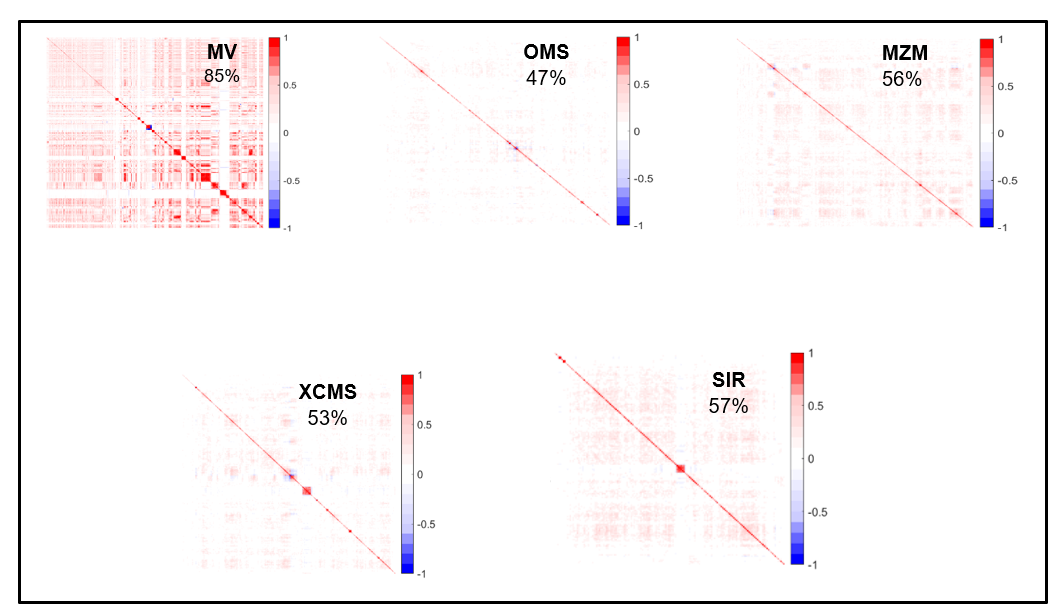


**Figure S7**: Multivariate Exploratory Data Analysis (MEDA) plots for data matrices retrieved from the five feature extraction tools, for the validation dataset.

**Table S10:** Explained across over 10 PCs for the five examined software tools at 70% sparsity

| PC | MV | MZM | OMS | SIR | XCMS |
| --- | --- | --- | --- | --- | --- |
| 1 | 16.9 | 8.9 | 7.2 | 11.5 | 8.7 |
| 2 | 10.3 | 6.8 | 4.6 | 5.3 | 6.8 |
| 3 | 4.9 | 2.4 | 2.6 | 2.9 | 4.1 |
| 4 | 3.4 | 1.7 | 2.2 | 2.2 | 2.2 |
| 5 | 2.6 | 1.7 | 2.2 | 2.1 | 1.9 |
| 6 | 2.3 | 1.6 | 2 | 2.1 | 1.9 |
| 7 | 2 | 1.5 | 1.9 | 2 | 1.8 |
| 8 | 1.8 | 1.4 | 1.7 | 1.8 | 1.8 |
| 9 | 1.5 | 1.4 | 1.6 | 1.7 | 1.6 |
| 10 | 1.1 | 1.2 | 1.6 | 1.8 | 1.6 |

**Table S11**: Explained across over 10 PCs for the five examined software tools at 90% sparsity

| PC | MV | MZM | OMS | SIR | XCMS |
| --- | --- | --- | --- | --- | --- |
| 1 | 5.9 | 3.7 | 2.9 | 4.7 | 4.3 |
| 2 | 4.5 | 3.3 | 2.9 | 3.7 | 3.7 |
| 3 | 4.3 | 1.5 | 1.7 | 3.3 | 3.3 |
| 4 | 2.3 | 1 | 1.4 | 1.9 | 1.5 |
| 5 | 2.3 | 1.1 | 1.4 | 1.7 | 1.4 |
| 6 | 2 | 1 | 1.3 | 1.7 | 1.3 |
| 7 | 2.2 | 0.9 | 1.3 | 1.4 | 1.1 |
| 8 | 1.4 | 0.9 | 1 | 1.5 | 1.0 |
| 9 | 1.3 | 0.8 | 1.1 | 1.2 | 1.0 |
| 10 | 0.9 | 0.8 | 1.1 | 1 | 1.0 |


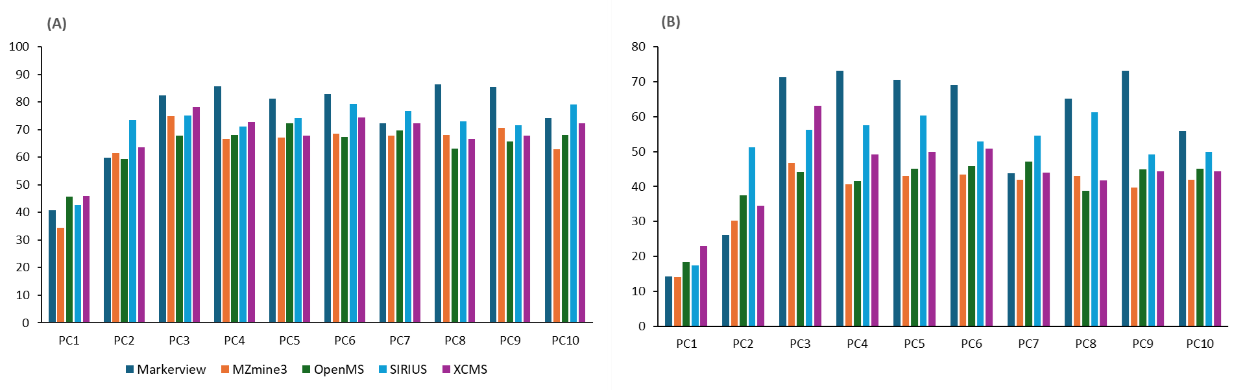

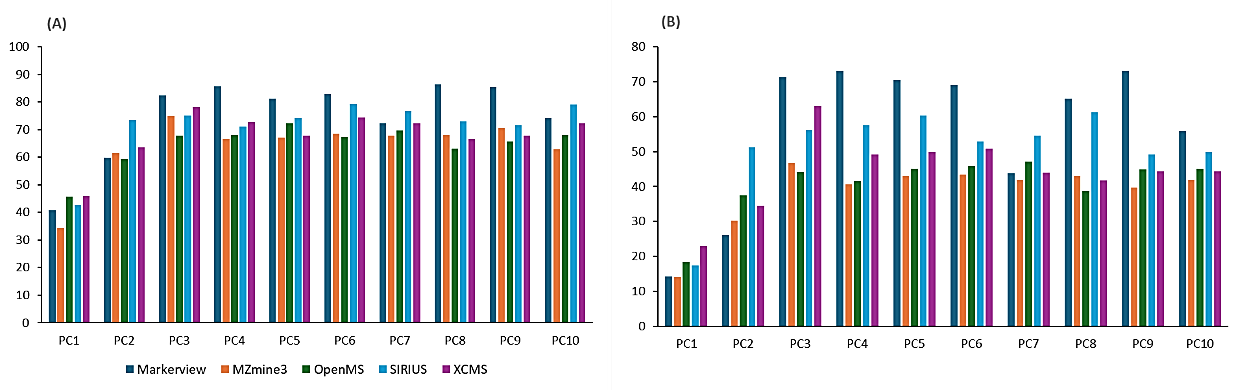


**Figure S8**: Proportion of retained explained variances for validation data set (I) in each latent space with regards to standard PCA counterpart in SPCA models, at ridge value=1 and (A) a Lasso penalty 0.7 and (B) 0.9, across five peak picking tools.

**Supplementary explanation for Feature ranking**

Figure 5 shows SPCA results for detecting Spill III (sulfamethoxazole) across five LC-HRMS software tools (calculated with delta = 10 for SIRIUS and delta = 1 for the remaining). Focusing on MarkerView, sulfamethoxazole and its sodium adduct are ranked as the second and third most influential features within PC6 under 90% sparsity. A grouping effect is evident, where correlated features (e.g., adducts and isotopologues) receive similar loading coefficients. However, the top-ranked variable (f470) corresponds to a matrix-related background component, aligning with a regular fluctuating compartment of PC6 trend. In both MZmine3 and OpenMS, sulfamethoxazole was successfully prioritized within PC6 and PC4, respectively. However, in each case, a background artifact, MZM274 in MZmine3 and OM87 in OpenMS, dominated the loadings. These features showed opposite loading signs compared to sulfamethoxazole, suggesting signal suppression at the time points when sulfamethoxazole appeared. These are referred to as spill-induced artifacts or features affected by spill events. Although misleading variables of MZM274/OM87 differ in retention time, their suppression patterns suggest a global ionization competition effect or issues related to peak misalignment. Notably, MZM274 corresponds to OM34 in OpenMS, highlighting a shared matrix-origin feature across tools. These findings illustrate how matrix effects, ion suppression, and software-specific feature extraction deteriorate the performance of SPCA models, mainly due to promoting artifact signals alongside true target trends. In SIRIUS, sulfamethoxazole was prioritized in PC10 only under stronger regularization (L2 = 10, 90% sparsity), enabling detection of this rare-event signal. Its delayed appearance, compared to earlier PCs in OpenMS and XCMS, is due to dominant variance from atrazine, epoxyconazole, and matrix-related spikes occupying earlier components. In contrast, XCMS clearly detected sulfamethoxazole in PC4—similar to OpenMS and without additional tuning. However, both tools exhibited high-loading artifact features among the top variables, confirming the persistent influence of matrix impact across platforms.

Figure S9 presents SPCA scores and loadings associated with detecting monotonic trends (2-TFMB, fluopyram, prometryn, absent from the Sirius dataset), primarily captured in PC2 across four software tools. The top panels illustrate loading distributions under 90% and 70% sparsity, showing how penalization affects feature selection. Score trajectories reveal clear upward or downward trends, broadly consistent with the spiked targets. Under the optimal Elastic Net penalty setting (Table 1), MarkerView tends to retain correlated and significant variables together, allowing both target compounds and their associated adducts or fragments to be prioritized simultaneously—an effect amplified by the absence of a feature componentization step. As a result, PC2 displays a smoother and more globally structured pattern (explaining 4.5% of variance), likely shaped by both correlated targets and (nonprioritized) residual drift. Generally, while PC2 reflects monotonic patterns, several matrix-related features are co-ranked with similar loading weights, indicating confounding from partially untreated sample drift. As a result, the observed PC2 trends represent a blend of true temporal signal and systematic background structure (which is clear for MZmine3, OpenMS and XCMS)—highlighting the challenge of isolating target-specific variance, when the IS-based normalization alone (or any other imperfect normalization method) may not sufficient to correct signal drift for all features. The cross-tool feature map comparison in Figure S9 confirmed that only 2-TFMB/Na+ adducts are consistently prioritized, with no false positive features shared across tools.

**MZmine3**

**OpenMS**

**XCMS**

**MarkerView**

**Fluopyram**

**Prometryn**

**Prometryn Na+**

**2-TFMB**

2-TFMB Na+

**2-TFMB**

2-TFMB

**Fluopyram**

**Prometryn**

**Prometryn**

**Fluopyram**

**2-TFMB**

**Fluopyram Na+**


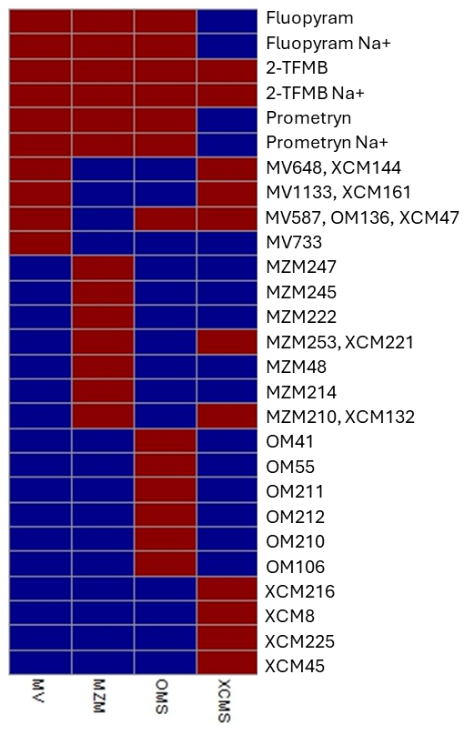


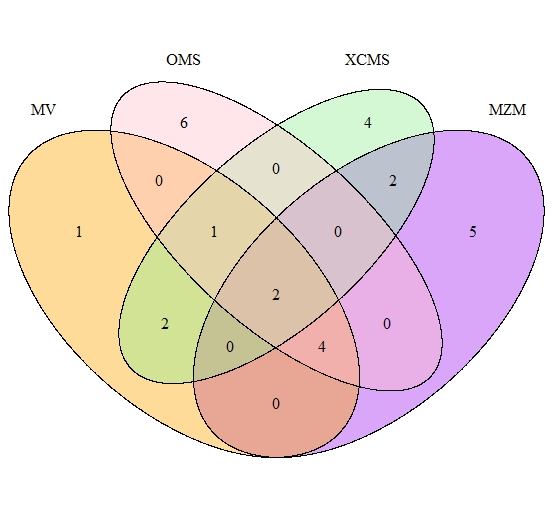


**MV: L2 = 10, L1=90%**

**OMS, MZM, XCMS: L2 =1, L1= 90%**

**XCMS: L2=1, L1= 70%**

**Figure S9:** *Comparison of the performance of the four evaluated software (loading and score plots, top features with highest loading values) for features exhibiting a downward/upward trend, as captured by SPCA. The heatmap and Venn diagram compare the common top 10 features. These targets were absent in the data matrix of SIRIUS*

## 4.3 Multivariate processing of real wastewater data: dataset (II)

To gain an initial overview of data structure, PCA was applied to the preprocessed datasets generated by OpenMS, MZmine3, and XCMS. The PC1–PC2 score plots (Figure S10) reveal broadly consistent sample clustering patterns across tools (two main subgroups of samples), with distinct highly leverage points (e.g., samples 3 and 4) consistently appearing in all three panels. While the orientation and spread of the points differ slightly, the core structure appears reproducible. Explained variance plots (bottom row) show a comparable rate of variance capture by the first few components across platforms. However, XCMS exhibits slightly higher residual variance (~56% after 10 PCs), which might be due to higher degree of redundant (or even true) features left in the residual matrix, whereas OpenMS and MZmine3 demonstrate more harmonized variance compression (~47% and ~49%, respectively). These differences may reflect differences in feature extraction stringency and filtering protocols used in each software.


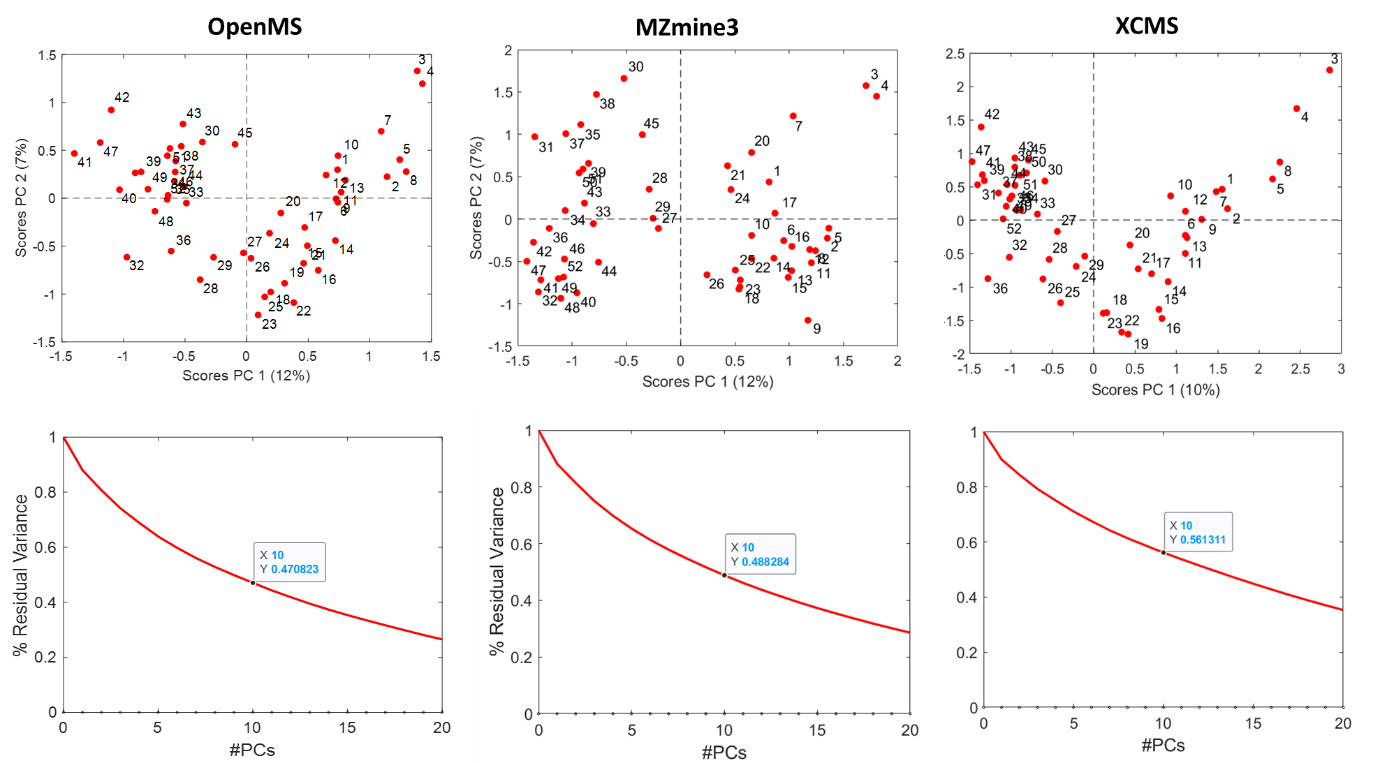


**Figure S10**: Scatter plots of PC scores (PCs 1-2) and the % residual variance across 20 PCs for data matrices retrieved from the three selected feature extraction tools, for real wastewater samples.


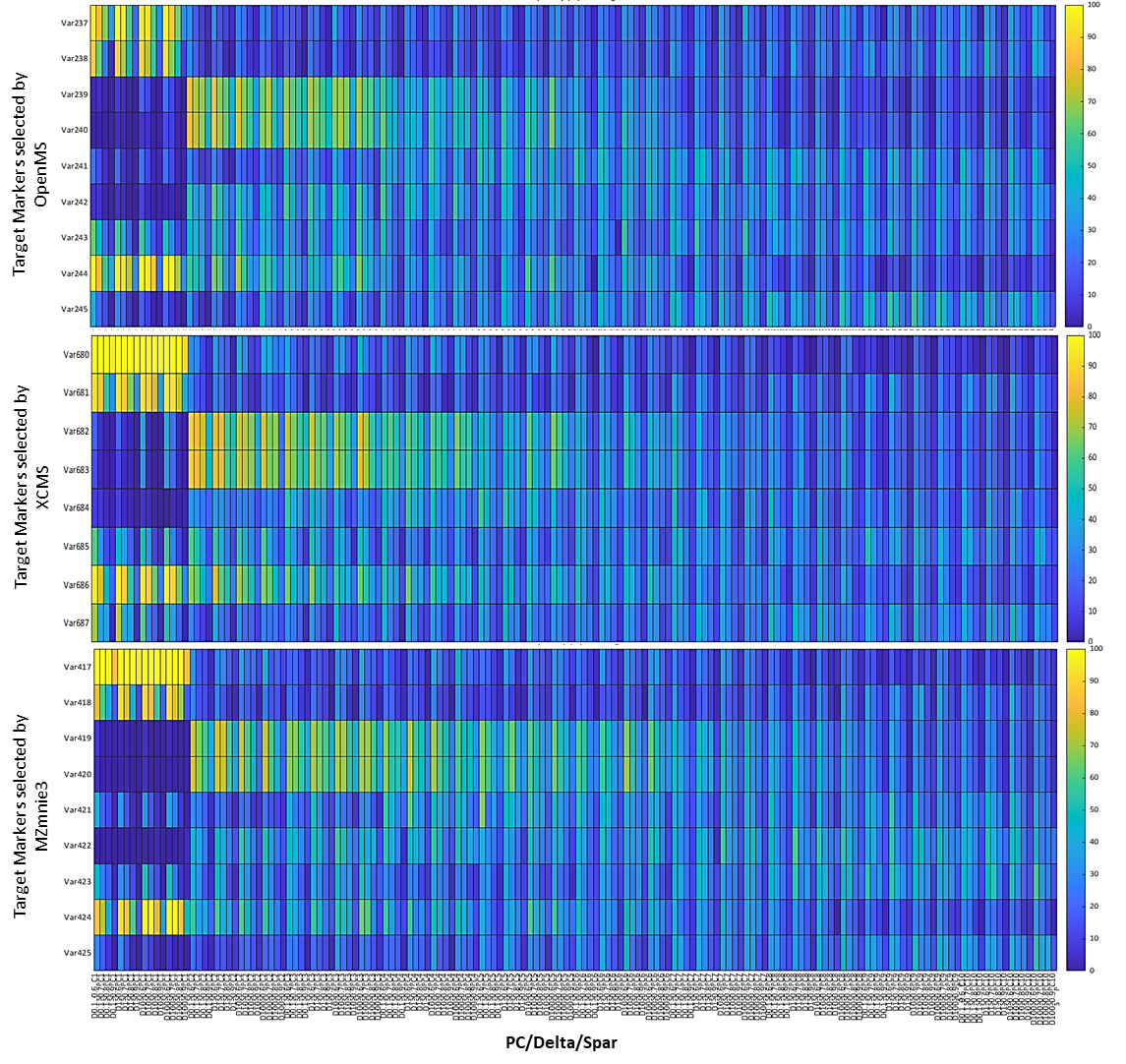


**Figure S11**: Selection frequency (%) for target markers in real wastewater samples. Each row represents a target feature, and columns reflect combinations of PC, delta, and sparsity (1440 total). Color intensity indicates selection frequency across bootstraps (0% = blue; 100% = yellow), summarizing feature robustness under varying SPCA conditions.


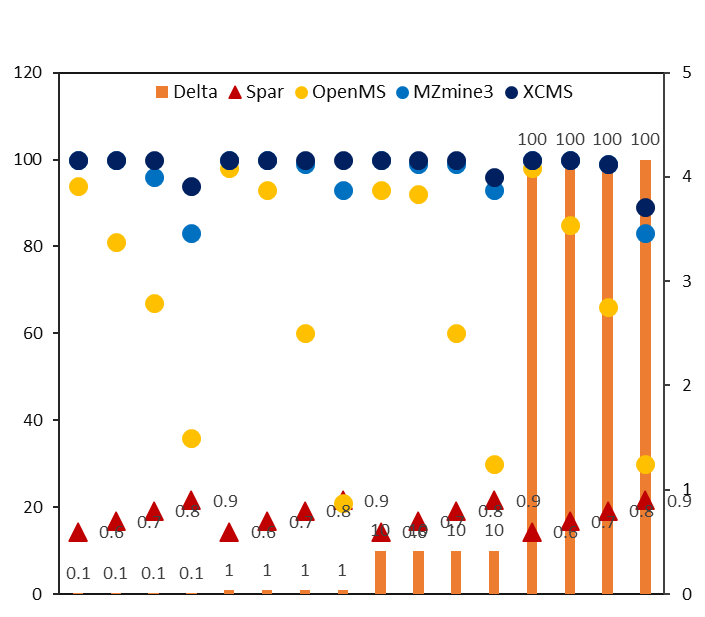


**Figure S12:** Representation of selection frequency for Picolinafen under differing tuning parameters across three software pipelines


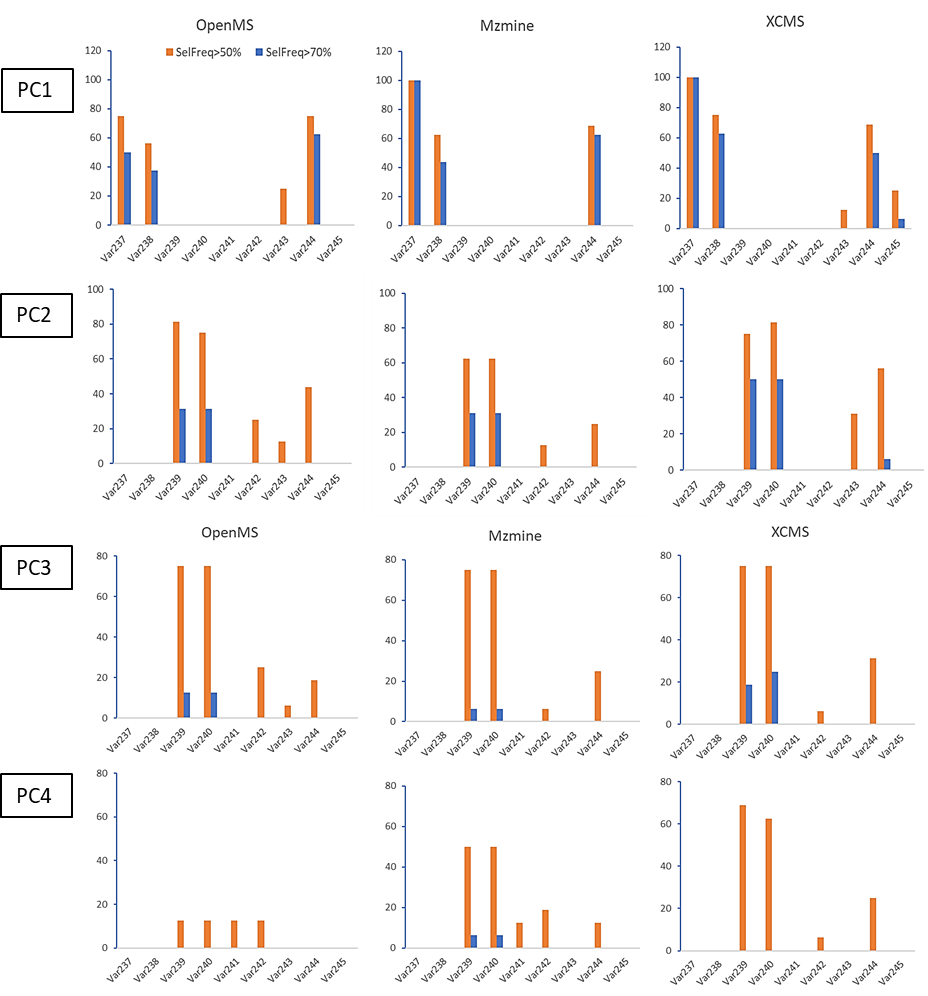


**Figure S13**: Proportion of selection frequency values exceeding 50% and 70% across 16 SPCA parameter settings for PCs 1-4, for each software-derived dataset


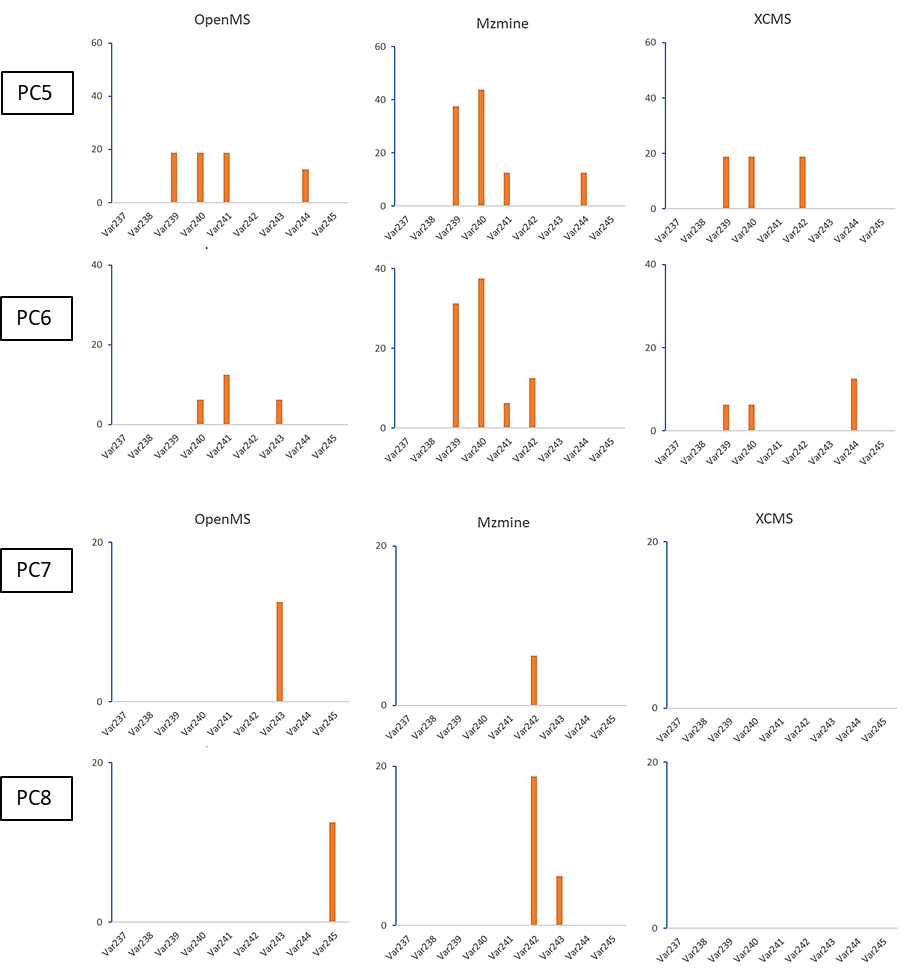


**Figure S14**: Proportion of selection frequency values exceeding 50% and 70% across 16 SPCA parameter settings for PCs 5-8, for each software-derived dataset

**Figures S13 and S14.** Target markers 237-240 show a pair-wise agreement between each pair of software tools, target marker Var244 (clothianidin) with right-sided spill started from day 39 consistently and globally achieved high stability of detection across tools (with the average proportion of selection frequency >70% equal to 58±7%), revealing its strong association with PC1 in all data types. One exception throughout this comparison is detection of target marker Var245 (atrazin, with a middle transient/periodic pattern, 13% exposure). This marker can be captured confidently only by XCMS with selection frequency >70% (ratio=6.2%) in the first PC. This marker was not detected in any latent spaces 1 to 8 using MZmine3 and only detected in PC8 using OpenMS (70%< frequency >50%). In addition, all pipelines indicate that Var241–243 play a modest role in shaping the latent space, given their lower selection frequencies (typically < 70%) across conditions. Detection efficiency of V242 (with 17.3% spill exposure and an overlapping pattern with V239-240) with a spread along various PCs (70%< frequency >50%), is showing more strong dependency to tuning parameters. Spill marker Var243 (exposure of 7.6%) was coherently detected with 70%< frequency >50% in PC1-2 across OpenMS and XCMS. In addition, both OpenMS and MZmine3 could confidently detect this profile in PCs 7 and 8. However, with a further connection to absolute loading values this spill could be detected only via OpenMS as a leading feature of the timeseries score 7 (see Figure S16). These differences primarily arise from how transient or sparsely occurring marker signals (e.g., narrow spills) are represented in the data. When such signals exhibit limited temporal persistence, the agreement of different software tools to consistently detect them diminishes. This is also the case for Var245 with (conditional) frequency >70% with 3% exposure, robustly detected only using XCMS. This inconsistency is mainly affected by the preprocessing methods used by different peak picking tools, which can significantly alter the data's variance structure, impacting both targeted and non-targeted features.


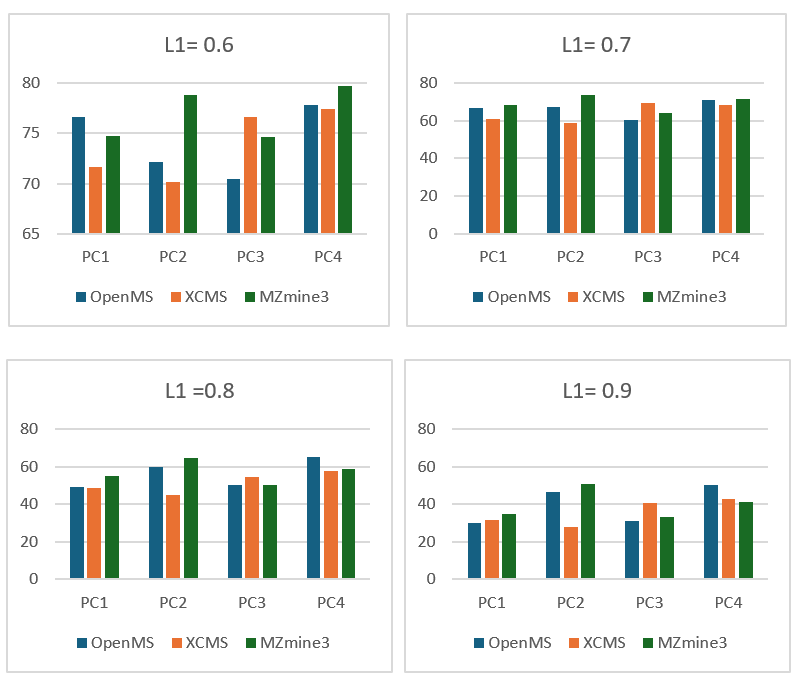


**Figure S15**: Proportion of retained explained variances for real wastewater samples in four latent spaces with regards to standard PCA counterpart in SPCA models, at ridge value=1 and L1 (lasso penalties) 0.6 to 0.9 across three selected peak picking tools.


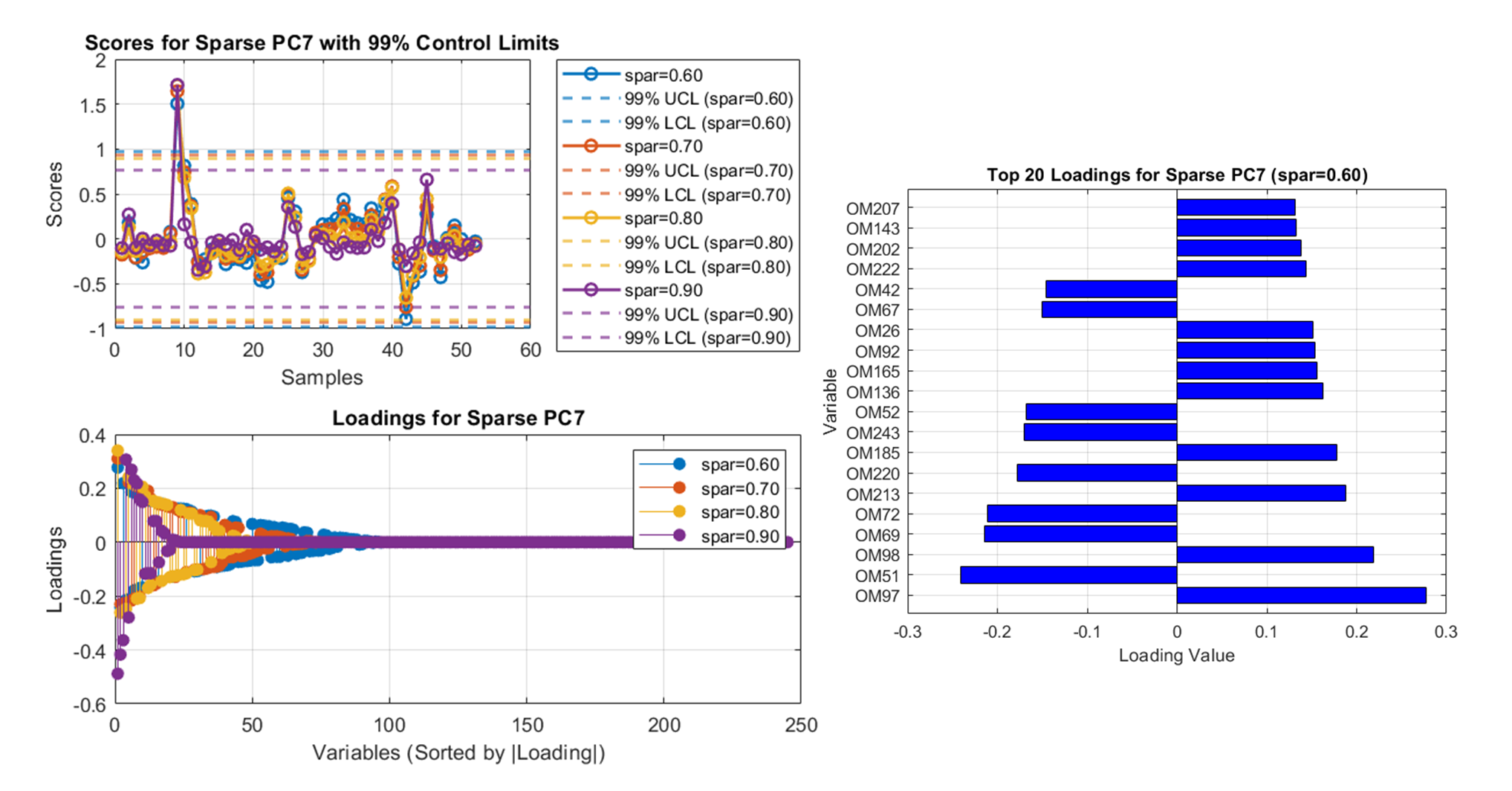


***Figure S16****:* *Score and loadings plots for PC7 at 60%- to 90% sparsity levels and L2 = 1, for OpenMS. The leading features with the highest loadings calculated for PC 7 at 60% sparsity are listed (Var243 is the target marker sulfamethoxazole).*

**Table S12:** Comparing selection stability and feature ranking of target markers using bootstrap SPCA under the two globally tuned sparsity parameters.

|  | **V237** | **V238** | **V239** | **V240** | **V241** | **V242** | **V243** | **V244** | **V245** |
| --- | --- | --- | --- | --- | --- | --- | --- | --- | --- |
| **Tuning: L1=0.7, L2=1** | | | | | | | | | |
| **PC** | 1 | 1 | 3 | 3 | 4 | 4 | 7 | 1 | 1 |
| **Bootstrap-Stability*** | 93/100/100 | 71/77/76 | 66/64/62 | 63/65/64 | 25/33/F** | 43/44/43 | 29/39/35 | 91/83/86 | 20/13/40 |
| **Abs.Loading Ranking*** | 18/6/1 | >20/>20/>20 | 1/2/9 | 2/4/11 | 11/5/F | >20/>20/4 | 14/>20/>20 | 14/>20/>20 | >20/>20/>20 |
| **Tuning: L1=0.6, L2=1** | | | | | | | | | |
| **Bootstrap- Stability*** | 98/100/100 | 87/90/92 | 71/70/75 | 71/70/74 | 34/50/F | 50/44/54 | 55/48(54***)/37 | 94/91/95 | 27/31/73 |
| **Abs.Loading Ranking** | 19/6/1 | >20/>20/>20 | 1/2/9 | 3/4/10 | 13/4/F | >20/>20/3 | 9/>20/>20 | 14>20/>20 | >20/>20/>20 |

*Results for OpenMS/MZMine3/XCMS

** Feature removed due to meeting near-zero variance (NZV) filtering criteria (exposure%=5.7).

***Detection in PC8


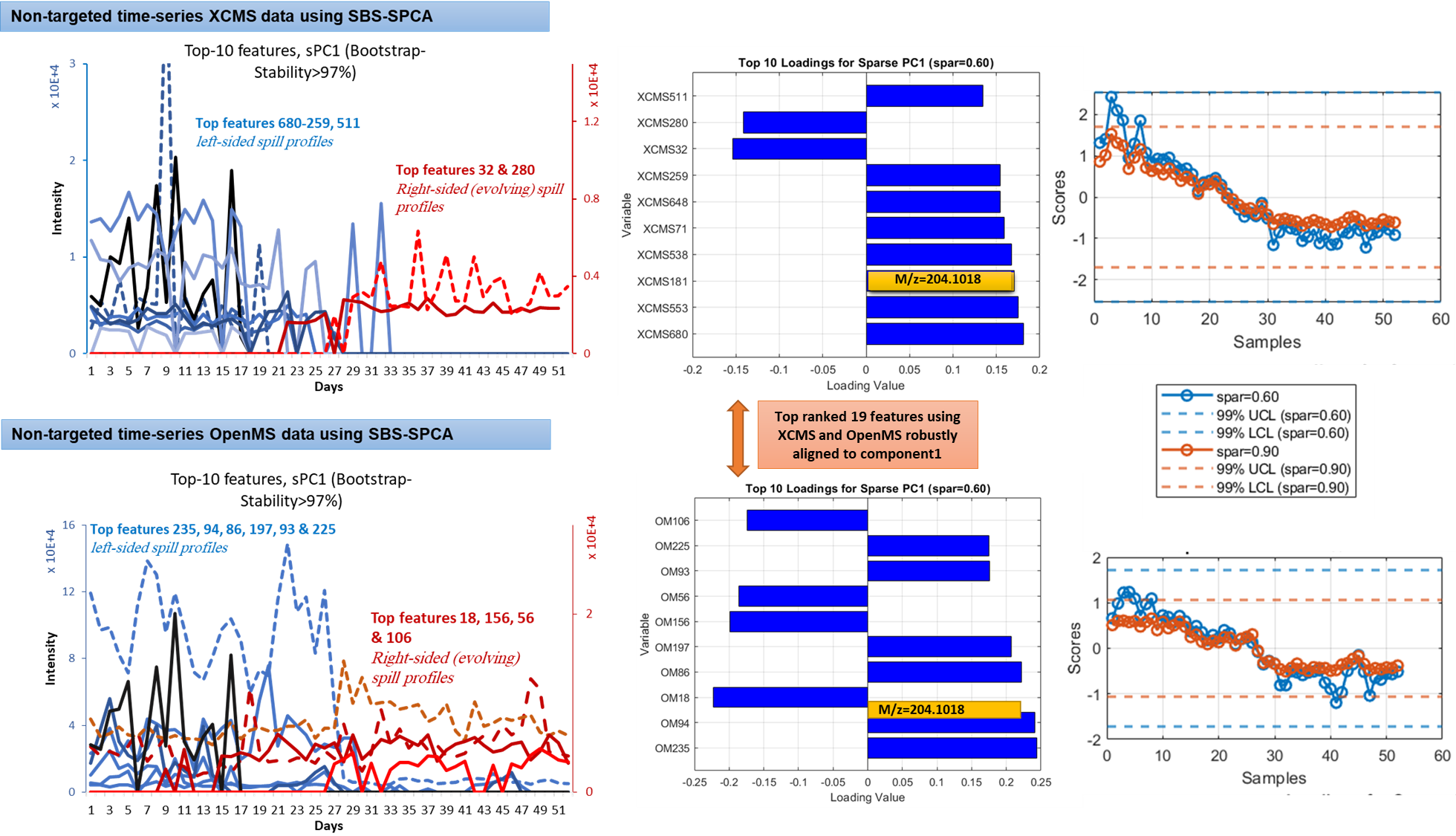


***Figure S17****: Application of SBS-SPCA on non-targeted OpenMS and XCMS time-series datasets. In this representative*  *case, top 10 features from Sparse PC1 (under optimal tuning L1= 60% and L2=1) were prioritized based on selection stability (>97%) and absolute loading ranks. Despite the high within-tool stability and strong loading values observed across both tools, only one feature (m/z = 204.1018) was shared between the top-ranked lists of OpenMS and XCMS, underlining the divergence in tool-specific rankings and the importance of cross-tool aggregation.*

**5.** **References**

1. Vosough M, Schmidt TC, Renner G (2024) Non-target screening in water analysis: recent trends of data evaluation, quality assurance, and their future perspectives. Anal Bioanal Chem. https://doi.org/10.1007/s00216-024-05153-8

2. Zou H, Hastie T, Tibshirani R (2006) Sparse Principal Component Analysis. J Comput Graph Stat 15:265–286. https://doi.org/10.1198/106186006X113430

3. Guerra-Urzola R, van Deun K, Vera JC et al. (2021) A Guide for Sparse PCA: Model Comparison and Applications. Psychometrika 86:893–919. https://doi.org/10.1007/s11336-021-09773-2

4. Mamouei M, Zhu Y, Nazarzadeh M et al. (2022) Investigating the association of environmental exposures and all-cause mortality in the UK Biobank using sparse principal component analysis. Scientific Reports 12:9239. https://doi.org/10.1038/s41598-022-13362-3

5. Ali SH, Cook T, Ewaid SH et al. (2023) Development of a Water Quality Index Using Sparse Principal Component Analysis for the Tigris River in Iraq. Water Resour 50:152–167. https://doi.org/10.1134/S0097807823010037

6. Myers OD, Sumner SJ, Li S et al. (2017) Detailed Investigation and Comparison of the XCMS and MZmine 2 Chromatogram Construction and Chromatographic Peak Detection Methods for Preprocessing Mass Spectrometry Metabolomics Data. Anal Chem 89:8689–8695. https://doi.org/10.1021/acs.analchem.7b01069

7. Pluskal T, Castillo S, Villar-Briones A et al. (2010) MZmine 2: Modular framework for processing, visualizing, and analyzing mass spectrometry-based molecular profile data. BMC Bioinformatics 11:395. https://doi.org/10.1186/1471-2105-11-395

8. Schmid R, Heuckeroth S, Korf A et al. (2023) Integrative analysis of multimodal mass spectrometry data in MZmine 3. Nat Biotechnol 41:447–449. https://doi.org/10.1038/s41587-023-01690-2

9. Tautenhahn R, Böttcher C, Neumann S (2008) Highly sensitive feature detection for high resolution LC/MS. BMC Bioinformatics 9:504. https://doi.org/10.1186/1471-2105-9-504

10. Helmus R, Laak TL ter, van Wezel AP et al. (2021) patRoon: open source software platform for environmental mass spectrometry based non-target screening. J Cheminform 13:1. https://doi.org/10.1186/s13321-020-00477-w

11. Sturm M, Bertsch A, Gröpl C et al. (2008) OpenMS - an open-source software framework for mass spectrometry. BMC Bioinformatics 9:163. https://doi.org/10.1186/1471-2105-9-163

12. Dührkop K, Fleischauer M, Ludwig M et al. (2019) SIRIUS 4: a rapid tool for turning tandem mass spectra into metabolite structure information. Nat Methods 16:299–302. https://doi.org/10.1038/s41592-019-0344-8
